# Intrinsic measurements of exciton transport in photovoltaic cells

**DOI:** 10.1038/s41467-019-09062-8

**Published:** 2019-03-11

**Authors:** Tao Zhang, Dana B. Dement, Vivian E. Ferry, Russell J. Holmes

**Affiliations:** Department of Chemical Engineering and Materials Science, University of Minnesota, Minneapolis, MN 55455 United States

## Abstract

Organic photovoltaic cells are partiuclarly sensitive to exciton harvesting and are thus, a useful platform for the characterization of exciton diffusion. While device photocurrent spectroscopy can be used to extract the exciton diffusion length, this method is frequently limited by unknown interfacial recombination losses. We resolve this limitation and demonstrate a general, device-based photocurrent-ratio measurement to extract the intrinsic diffusion length. Since interfacial losses are not active layer specific, a ratio of the donor- and acceptor-material internal quantum efficiencies cancels this quantity. We further show that this measurement permits extraction of additional device-relevant information regarding exciton relaxation and charge separation processes. The generality of this method is demonstrated by measuring exciton transport for both luminescent and dark materials, as well as for small molecule and polymer active materials and semiconductor quantum dots. Thus, we demonstrate a broadly applicable device-based methodology to probe the intrinsic active material exciton diffusion length.

## Abstract (web summary)

Zhang et al. develop a device-based method to probe intrinsic exciton transport in photovoltaic cells. The broad utility of this method is demonstrated by measuring exciton transport for both luminescent and dark organic semiconductors as well as semiconductor quantum dots.

## Introduction

Excitons are Coulombically-bound electron-hole pairs that serve as energetic intermediates in semiconductors. In many emerging semiconductor materials, the exciton and its properties feature prominently in determining device applications due to large binding energies that stabilize the exciton against thermally-driven dissociation^1–8^. Indeed, the high stability of the exciton has driven much research into the design of active materials and device architectures for organic light-emitting devices and organic photovoltaic cells (OPVs)^9–15^. Recently, there has also been growing interest in manipulating exciton transport at semiconductor heterointerfaces for devices like excitonic transistors^16–18^. For OPVs, in particular, excitons must migrate to a dissociating electron donor–acceptor (D-A) interface, making exciton transport a critical step towards efficient photoconversion^19–24^. Consequently, substantial previous work has been directed at both the measurement and engineering of the exciton diffusion length (*L*_D_). These efforts are essential as the scale of *L*_D_ dictates the optimal active layer thickness and domain size of D–A blends in planar (PHJ) and bulk heterojunction (BHJ) OPVs, respectively^25–29^.

Probing exciton migration often relies on tracking the end-of-life products of excitons, including photons from exciton recombination and charge carriers from exciton dissociation^30,31^. For luminescent materials, *L*_D_ can be determined from a steady-state or time-resolved photoluminescence (PL)-based measurement^22,32,33^. Emitted photons reflect excitons that fail to reach the dissociating interface. While capable of yielding a materials-relevant value of *L*_D_, this method is only sensitive to diffusive states that can decay radiatively and therefore cannot be applied to materials forming weakly emissive or dark excitons^34–36^. Since many high-performing active materials are weakly luminescent or dark^7,12,35,37,38^, it is essential to develop more general means to accurately probe exciton transport in working devices.

A more general approach to extract *L*_D_ is by fitting the measured photocurrent (i.e., external quantum efficiency) spectrum of a bilayer OPV^39–41^. Device-based methods are attractive as they probe the *L*_D_ in a practical environment, as opposed to the often idealized structures utilized for PL measurements. This method is equally applicable to all excitons regardless of photoluminescence efficiency. Photoconversion can be considered in terms of four component processes, each with its own efficiency, namely light absorption (*η*_A_), exciton diffusion and dissociation (*η*_D_), charge transfer (CT) state separation (*η*_CS_), and free carrier collection (*η*_FC_). As such, with knowledge of *η*_CS_ and *η*_FC_, coupled with an optical transfer matrix model for *η*_A_, *η*_D_ and *L*_D_ can be extracted by fitting with a diffusion equation. In practice, the values of *η*_CS_ and *η*_FC_ are not known, and frequently taken as unity or assumed to be fixed with changes in device architecture^39,42–45^. Thus, the extracted value of *L*_D_ is a lower bound to the actual, materials-specific *L*_D_. This limitation, coming from unknown geminate and non-geminate recombination losses, must be overcome in order to extract intrinsic, materials-specific values of *L*_D_.

Recently, we reported a technique based on transient photovoltage to overcome the non-geminate recombination limitation on extracting *L*_D_ from OPVs^46,47^. Photovoltage can be quantitatively translated into the number of free carriers stored within the device. For timescales much shorter than the carrier lifetime, the free carrier generation rate can be extracted from the initial photovoltage rise induced by illumination. The previously unknown *η*_FC_, therefore, can be quantitively measured by comparing the results of photovoltage and photocurrent measurements. Interestingly, *η*_FC_ is found to be near-unity at short-circuit for many PHJ systems. Consequently, an inability to quantify geminate recombination loss is the primary factor frustrating accurate extraction of *L*_D_ in devices.

Despite the presence of unknown recombination losses, it is possible to measure the intrinsic *L*_D_ from a device without the need to assume a value for *η*_CS_. Previously, Vandewal et al. have shown that relaxed interfacial CT states (lowest energy) are the dominant source for free carrier generation in a wide range of D–A systems^48^. This conclusion is further confirmed by recent work by Kurpiers et al.^49^. The value of *η*_CS_ is thus independent of whether a carrier originates from the donor or acceptor exciton. As such, a ratio of the donor and acceptor internal quantum efficiencies (*η*_IQE_) cancels this unknown quantity and depends only on the exciton harvesting efficiencies (*η*_D_) at short-circuit:

1 $$\frac{{\eta _{{\mathrm{IQE}}}^{\mathrm{D}}}}{{\eta _{{\mathrm{IQE}}}^{\mathrm{A}}}} = \frac{{\eta _{\mathrm{D}}^{\mathrm{D}}\eta _{{\mathrm{CS}}}}}{{\eta _{\mathrm{D}}^{\mathrm{A}}\eta _{{\mathrm{CS}}}}} = \frac{{\eta _{\mathrm{D}}^{\mathrm{D}}}}{{\eta _{\mathrm{D}}^{\mathrm{A}}}}$$

In order to model *η*_D_, exciton diffusion is treated with a one-dimensional steady-state diffusion equation:

2 $$0 = D\frac{{\partial ^2n\left( x \right)}}{{\partial x^2}} - \frac{{n\left( x \right)D}}{{L_{\mathrm{D}}^2}} + G\left( x \right) - n\left( x \right)k_{\mathrm{F}}\,{\mathrm{with}}\,L_{\mathrm{D}} = \sqrt {D\tau }$$

where *n* is the exciton density, *D* is the diffusion coefficient, *τ* is the exciton lifetime, and *G* is the optical exciton generation rate calculated by transfer matrix modeling. For systems with a spectral overlap of donor emission and acceptor absorption, the rate of donor–acceptor Förster energy transfer (rate constant: *k*_F_) must be included. From the steady-state exciton density profile *n*(*x*), the *η*_D_ can be determined as:

3 $$\eta _{\mathrm{D}} = 1 - \frac{{{\int} {n(x)/\tau {\mathrm{d}}x} }}{{{\int} {G\left( x \right){\mathrm{d}}x} }}$$

By fitting the *η*_D_ ratio in eq. (1) as a function of active layer thickness, *L*_D_ values for the donor and acceptor materials can be extracted simultaneously.

**Fig. 1:**
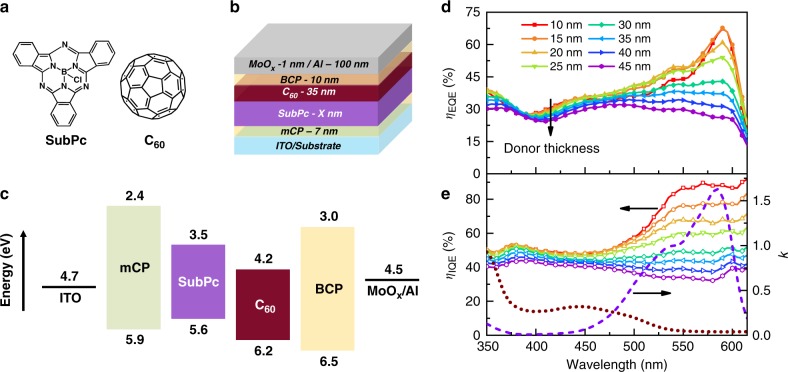
Device architectures and quantum efficiency spectra for SubPc-C_60_ planar OPVs. **a** Molecular structure of SubPc and C_60_. **b** Device architecture for SubPc-C_60_ planar OPVs. The SubPc thickness X varies from 10 nm to 45 nm. **c** Energy-level diagram for the devices in **b**. **d** The *η*_EQE_ spectra measured at short-circuit as a function of SubPc layer thickness. **e** The *η*_IQE_ spectra calculated by dividing the *η*_EQE_ spectra in **d** by the *η*_A_ calculated using a transfer matrix model. The extinction coefficients (*k*) of SubPc (purple dash line) and C_60_ (brown dot line) are also shown

In this work, we apply the photocurrent-ratio method to extract *L*_D_ for both luminescent and dark organic semiconductors, including both small molecule and polymer active materials. We further demonstrate the broad utility of this approach by also extracting *L*_D_ for thin films of colloidal, inorganic semiconductor quantum dots. In order to compare the results of the photocurrent-ratio method to well-established photoluminescence quenching methods, we first consider the archetypical donor–acceptor pairing of boron subphthalocyanine chloride (SubPc) and C_60_ (Fig. 1a), where both active materials are luminescent. This system shows negligible non-geminate recombination loss at short-circuit^50,51^, and prior work suggests that free carriers are likely generated from a single relaxed CT state^52^, allowing eq. (1) to be applied. It is worth noting that in devices limited by non-geminate recombination, *η*_FC_ can be measured using transient photovoltage, leading to a modified, but equally useful form of eq. (1). For this luminescent pairing, we demonstrate that the values of *L*_D_ extracted using our device-based method are in excellent agreement with those extracted using conventional PL-based measurements. Importantly, we further show that our device-based method yields the intrinsic material *L*_D_ even for devices that suffer from very low efficiency.

## Results

### Extracting *L*_D_ from a ratio of internal quantum efficiencies

To accurately measure *L*_D_ in bilayer OPVs, it is important to ensure that the only mechanism for exciton dissociation is at the donor–acceptor interface. Quenching at other interfaces or via bulk-ionization will lead to errors in the extracted *L*_D_. Figure 1b shows the SubPc-C_60_ PHJ OPV used for the measurement of *L*_D_. The wide energy gap organic semiconductors 1,3-bis(carbazol-9-yl)benzene (mCP) and bathocuproine (BCP) are employed as exciton blocking layers (EBLs) to prevent exciton quenching at active layer-electrode interfaces. The exclusion of quenching at the electrodes is critical for accurate measurements of *L*_D_ as exciton loss is observed when SubPc is in direct contact with ITO or the commonly used buffer layer MoO_x_ (Supplementary Figure 1)^53^. A 1-nm-thick layer of MoO_x_ is used to increase the cathode work function and reduce the built-in electric field (*E*_bi_) to exclude efficient exciton bulk-ionization processes at short-circuit^54^. This is especially important for the SubPc-C_60_ system since both materials have been observed to show efficient free carrier generation in Schottky OPVs^55,56^. While the application of a forward bias can also be used to reduce *E*_bi_, the resulting carrier injection into the device could lead to exciton quenching, and an underestimate of *L*_D_. It is worth noting that while a reduced *E*_bi_ could hinder the separation of relaxed CT states at the donor–acceptor interface, we will demonstrate that this does not impact our measurement provided *η*_CS_ is identical for charges originating from both active materials.

**Fig. 2:**
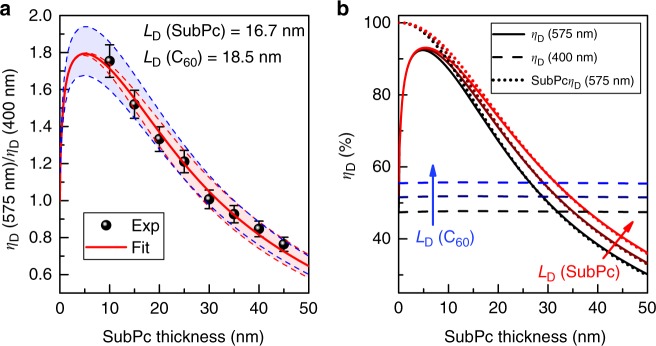
Extracting *L*_D_ from the thickness dependence of the diffusion efficiency ratio. **a** Diffusion efficiency ratio (*λ* = 575 nm to *λ* = 400 nm) as a function of SubPc layer thickness with the corresponding exciton diffusion length (*L*_D_) extracted from the fit (solid red line) to the data. Red (blue) dash lines are simulated diffusion efficiency ratio curves for a 10% change in extracted SubPc or C_60_ *L*_D_ while keeping the counterpart *L*_D_ fixed. Error bars of diffusion efficiency ratio were determined based on the standard deviation of measured devices. **b** Solid lines are the diffusion efficiency at *λ* = 575 nm as a function of SubPc layer thickness for different SubPc *L*_D_ ( = 15.0/16.7/18.3 nm) and fixed C_60_ *L*_D_ = 18.5 nm. Similarly, the horizontal dash lines are the diffusion efficiency at *λ* = 400 nm for different C_60_ *L*_D_ ( = 16.7/18.5/20.3 nm) and fixed SubPc *L*_D_ = 16.7 nm. The diffusion efficiencies of SubPc at *λ* = 575 nm are also plotted as dot lines

Figure 1d shows external quantum efficiency (*η*_EQE_) spectra for SubPc-C_60_ PHJ devices as a function of SubPc donor-layer thickness with the acceptor and buffer layer thicknesses held fixed. The *η*_A_ of the active materials calculated using a transfer matrix formalism (Supplementary Figure 2) allows the *η*_EQE_ spectra to be converted to *η*_IQE_ spectra (Fig. 1e)^41^. To ensure the accuracy of the simulated *η*_A_, the result from transfer matrix modeling is checked against experimentally determined device reflectivity (through the anode, reflecting off the cathode) measured at an incident angle of 15° to the substrate normal. The simulated 1-reflectivity (*R*) spectra (absorption of the device stack) agree well with experimental results as a function of donor thickness (Supplementary Figure 3). For the device with a donor thickness of 10 nm, the maximum *η*_EQE_ (at *λ* = 590 nm, SubPc absorption) exceeds 65% and the corresponding *η*_IQE_ is about 85%. This indicates that both exciton harvesting and charge collection are efficient in this device despite the reduced *E*_bi_. From the extinction coefficients (k) of SubPc and C_60_, spectral regions of dominant absorption for both donor and acceptor can be isolated. Accordingly, for the construction of the *η*_IQE_ ratio, the individual *η*_IQE_ of the donor and acceptor are extracted from *λ* = 575 nm (primarily SubPc absorption) and *λ* = 400 nm (primarily C_60_ absorption), respectively. Consistent with eq. (1), a *η*_D_ ratio (*λ* = 575 nm to *λ* = 400 nm) is calculated as a function of SubPc thickness (Fig. 2a), which will be fit for *L*_D_.

By fitting the *η*_D_ ratios in Fig. 2a, we simultaneously extract *L*_D_ values for SubPc and C_60_ of *L*_D_ = (16.7 ± 1.7) nm and *L*_D_ = (18.5 ± 1.9) nm, respectively. Due to the spectral overlap of SubPc PL emission and C_60_ absorption, donor–acceptor Förster energy transfer is included when using eq. (2). We have previously measured the SubPc-C_60_ Förster radius (*R*_0_) to be 2.1 nm, consistent with the value of 2.1 nm calculated using Förster theory^32^. This value is short compared to the intrinsic *L*_D_ of SubPc, and hence does not significantly impact our measurement of *L*_D_ as diffusion is the dominant mechanism for harvesting in SubPc. As only the donor thickness is varied, the *L*_D_ of the donor and acceptor have different roles in determining the overall behavior of the *η*_D_ ratio as thickness changes (dash lines in Fig. 2a). For example, if the SubPc *L*_D_ is varied by 10% (C_60_ *L*_D_ fixed), the resulting *η*_D_ ratios converge at small thickness while diverging at larger thickness, varying the shape of the dependence on thickness. Similar changes in the C_60_ *L*_D_ (10%, SubPc *L*_D_ fixed) lead only to changes in the magnitude of the *η*_D_ ratio leaving the shape unchanged. To show this more explicitly, the values of *η*_D_ at *λ* = 575 nm and 400 nm are plotted as a function of donor thickness in Fig. 2b.

The observation of a constant acceptor *η*_D_ with increasing donor thickness offers some inherent advantages. First, the *L*_D_ of the donor and acceptor can be fit independently, as the shape of the *η*_D_ ratio (or normalized *η*_D_ ratio) only depends on donor *L*_D_. As such, any non-ideality in acceptor *L*_D_ measurement, such as exciton loss at the acceptor-cathode interface, would not reduce the accuracy of donor *L*_D_ provided free carriers are still only generated at the D–A interface. In addition, the relative change in *η*_CS_ with donor thickness is directly reflected by the acceptor *η*_IQE_. Accordingly, the decrease in *η*_IQE_ (400 nm) with SubPc thickness (Fig. 1e) indicates that a thicker donor layer leads to a lower *η*_CS_. This is likely due to an increase in bulk resistance of the donor layer that consumes more built-in voltage and thus reduces the electric field strength at D–A interface^57^.

### Photoluminescence-based *L*_D_ measurements

**Fig. 3:**
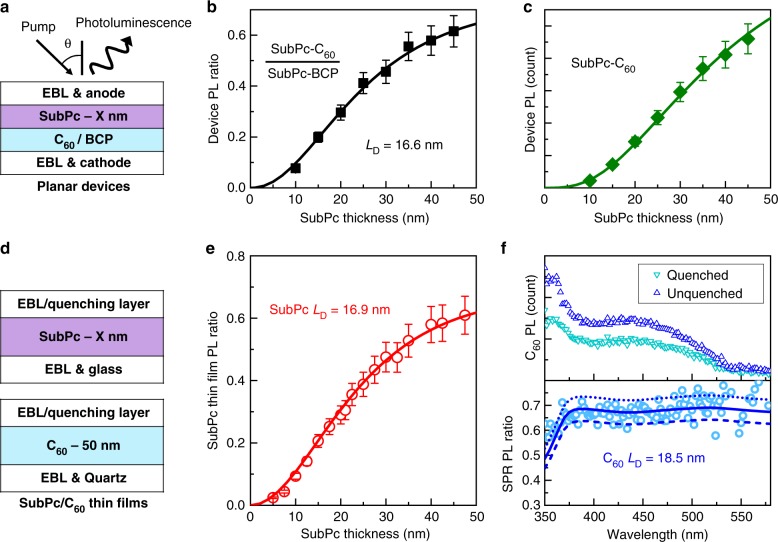
Photoluminescence-based measurements of exciton diffusion length. **a** OPVs used for the extraction of *L*_D_ via photoluminescence (PL) quenching having the structure: ITO/7 nm mCP/X ( = 10–45) nm SubPc/35 nm C_60_ (quenched) or BCP (unquenched)/10 nm BCP/1 nm MoO_x_/100 nm Al. The quenched samples are the same set of devices used for internal quantum efficiency measurements. **b** PL ratio defined in eq. (5) versus SubPc thickness. **c** SubPc PL intensity of the quenched samples versus SubPc thickness. The solid line is fit of the data using eq. (4). Error bars reflect the run-to-run variation in PL intensity (standard deviation). **d** Architectures for conventional thin-film PL ratio *L*_D_ measurements of SubPc and C_60_. SubPc architectures have the structure: glass/10 nm mCP/X ( = 5.0–47.5) nm SubPc/10 nm HATCN (quenched) or mCP (unquenched) while C_60_ architectures have the structure: quartz/10 nm BCP/50 nm C_60_/10 nm HATCN (quenched) or BCP (unquenched). **e** The PL ratio of SubPc films as a function of SubPc thickness. **f** Excitation spectra (upper panel) for the quenched and unquenched C_60_ films at an emission wavelength of 750 nm. The resulting spectrally resolved (SPR) PL ratio versus pump wavelength is shown in lower panel. SubPc films and devices are all pumped with *λ* = 500 nm light. The incident angle is 70° for all incident light. Error bars for PL ratios are propagated from measured spectra and are proportional to the standard deviation in PL intensity measured multiple times

To determine whether the *L*_D_ extracted from the *η*_IQE_ ratio is intrinsic, conventional PL-based measurements are carried out for both SubPc and C_60_. In many previous studies, *η*_EQE_-based measurements of *L*_D_ have not been in agreement with those extracted from PL due to geminate recombination losses^46,50^, which are in general not negligible. If the recombination limitations are fully overcome in the method described here, these measurements should yield the same *L*_D_. For luminescent SubPc, the intrinsic *L*_D_ is extracted using thickness-dependent PL quenching and compared to the result obtained using the photocurrent-ratio method. In order to make a proper comparison, PL-quenching measurements are carried out on the identical device architecture used to measure *L*_D_ via *η*_IQE_ (Fig. 3a). Light having a wavelength of *λ* = 500 nm is incident through the ITO anode at an angle θ of 70°. As the emitted photons represent the excitons that fail to reach D–A interface, the PL intensity can be expressed as:

4 $${\mathrm{PL}} = \eta _{\mathrm{A}}(1 - \eta _{\mathrm{D}})(I_{{\mathrm{in}}}\eta _{{\mathrm{PL}}}\eta _{{\mathrm{det}}})$$

where *I*_in_ is the incident photon flux, *η*_PL_ is the photoluminescence efficiency, *η*_det_ is the detection efficiency for all the emitted photons. Typically, *L*_D_ is extracted by fitting a ratio of the integrated PL of a quenched sample (Q) and an unquenched sample (UQ). This PL ratio reflects the number of recombining excitons (i.e., not collected at the quenching interface) under steady-state:

5 $$\frac{{{\mathrm{PL}}^{\mathrm{Q}}}}{{{\mathrm{PL}}^{{\mathrm{UQ}}}}} = \frac{{\eta _{\mathrm{A}}^{\mathrm{Q}}(1 - \eta _{\mathrm{D}}^{\mathrm{Q}})(I_{{\mathrm{in}}}\eta _{{\mathrm{PL}}}\eta _{{\mathrm{det}}})}}{{\eta _{\mathrm{A}}^{{\mathrm{UQ}}}(I_{{\mathrm{in}}}\eta _{{\mathrm{PL}}}\eta _{{\mathrm{det}}})}} = \frac{{\eta _{\mathrm{A}}^{\mathrm{Q}}(1 - \eta _{\mathrm{D}}^{\mathrm{Q}})}}{{\eta _{\mathrm{A}}^{{\mathrm{UQ}}}}}$$

The *η*_A_ and *η*_D_ can be simulated using a transfer matrix formalism and a diffusion model as previously described. Here, the SubPc-C_60_ PHJ devices in Fig. 1b are used as quenched samples, with C_60_ acting as the quencher. A set of unquenched samples (*η*_D_ = 0) are fabricated by replacing acceptor C_60_ with the exciton blocking material BCP. The resulting PL ratio is plotted in Fig. 3b and fit as a function of SubPc thickness, yielding a SubPc *L*_D_ of (16.6 ± 2.0) nm. Clearly, the photocurrent-ratio and PL-based measurements conducted on the same set of devices show excellent agreement for the extracted *L*_D_. This agreement suggests that the charge recombination present in the device-based measurement does not limit our ability to extract *L*_D_.

To further verify the extracted value of *L*_D_, we use the *L*_D_ determined by the PL ratio to simulate the PL of quenched devices with eq. (4). Figure 3c shows the integrated device PL of SubPc-C_60_ PHJ devices as a function of SubPc thickness. Taking the prefactor (*I*_in_*η*_PL_*η*_det_) as independent of SubPc thickness, the experimental results are in good agreement with the simulation using *L*_D_ = 16.6 nm for SubPc.

In most reports of PL-based measurements of *L*_D_, actual device architectures are not utilized, replaced instead with simple film stacks. In this configuration, exciton dissociation due to bulk-ionization is negligible due to the absence of electrodes. If bulk-ionization in SubPc is negligible for the devices in Fig. 1b, *L*_D_ values for SubPc extracted from device and thin-film PL should agree. As shown in Fig. 3d, SubPc thin films are deposited on a 10-nm-thick mCP/glass substrate to ensure the SubPc film morphology is similar to the device-based measurement. The quenched films are capped with a 10-nm-thick layer of 1,4,5,8,9,11-hexaazatriphenylene hexacarbonitrile (HATCN) while the unquenched films are capped with a 10-nm-thick layer of mCP^58^. Based on the thickness-dependent PL ratio of Fig. 3e, an intrinsic *L*_D_ of (16.9 ± 2.0) nm is determined for SubPc. This value is larger than previous reports of the SubPc *L*_D_, which is around 10 nm^32,59,60^. However, previous studies typically deposit SubPc directly on an inorganic substrate such as glass as opposed to a wide energy gap organic buffer layer. By removing the 10-nm-thick mCP layer beneath the SubPc for unquenched samples, we observe a reduction in *η*_PL_, suggesting a difference in the morphology of SubPc on different substrates.

For weakly luminescent C_60_, a thin layer can result in a very low signal to noise ratio, which increases error for the extraction of *L*_D_. As such, we employ a 50-nm-thick C_60_ layer and use spectrally resolved (SPR) PL quenching instead of thickness-dependent PL quenching^59^. We also use quartz as the substrate to further reduce the background noise. Figure 3f shows PL excitation spectra for the quenched and unquenched C_60_ films at an emission wavelength of *λ* = 750 nm. The PL ratio is fit as a function of incident excitation wavelength, yielding an intrinsic *L*_D_ of (18.5 ± 3.0) nm for C_60_. The excellent consistency of results between thin-film and device-based measurements further confirm that an intrinsic *L*_D_ can be accurately measured using the *η*_IQE_-based approach reported here. This agreement also suggests that there are no other diffusive dark states in SubPc and C_60_ that contribute to photocurrent since the photocurrent-ratio measurement probes both dark and emissive excitons while the PL-based measurement is only sensitive to emissive states.

### Relaxation losses for high-energy excited states

**Fig. 4:**
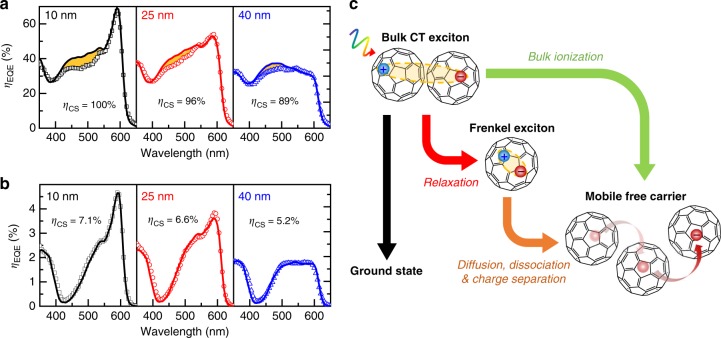
Simulation of external quantum efficiency for extraction of recombination loss. **a** External quantum efficiency spectra for SubPc-C_60_ planar OPVs as a function of SubPc thickness (10, 25, and 40 nm). Experimental results are shown in symbols while solid lines are simulated spectra. **b** The experimental (symbols) and simulated (solid lines) external quantum efficiency spectra of SubPc-NPD planar OPVs versus SubPc thickness. **c** Schematic of free carrier generation in C_60_ from a photoexcited bulk CT exciton. The bulk CT excitons absorb over the wavelength range of *λ* = 410 to 550 nm, while the low energy Frenkel excitons correspond to absorption at *λ* greater than 550 nm

With the *L*_D_ of SubPc and C_60_ extracted from device photocurrent spectra shown to be intrinsic, materials-relevant quantities, these values can be used to accurately simulate exciton diffusion and recombination losses in OPVs. The unknown geminate recombination loss (*η*_CS_) that was circumvented in eq. (1) by taking a ratio of *η*_IQE_ values can now be calculated as the ratio of *η*_IQE_ to the calculated *η*_D_. Figure 4a shows the simulated *η*_EQE_ spectra for SubPc-C_60_ PHJ devices using the *L*_D_ extracted in Fig. 1b, and the extracted *η*_CS_. The simulated *η*_EQE_ spectra agree well with experimental results except for the wavelength range of *λ* = 410–550 nm. The observed *η*_EQE_ overestimation indicates the presence of a photocurrent loss pathway prior to exciton diffusion, or stated differently, the exciton relaxation yield is not unity. It also worth noting the clear reduction in *η*_CS_ with increasing donor layer thickness, likely reflecting a reduction in *E*_bi_.

To identify whether the exciton relaxation loss is from SubPc or C_60_, a second donor–acceptor system of SubPc-NPD is examined where SubPc serves as the electron acceptor (or hole donor)^7^. To ensure that the SubPc morphology is unchanged, an inverted device architecture is employed (ITO/7 nm mCP/X nm SubPc/35 nm NPD/10 nm mCP/3 nm MoO_x_/100 nm Al) so that SubPc is still deposited on mCP. In these devices, the electron acceptor SubPc has low electron mobility, which can lead to significant recombination losses due to slow CT state separation^61^. As shown in Supplementary Figure 4, the SubPc *η*_IQE_ (from *λ* = 525 nm) and NPD *η*_IQE_ (from *λ* = 385 nm) are both very low (less than 8%). By fitting the *η*_D_ ratio as a function of SubPc thickness, an *L*_D_ of (15.2 ± 2.0) nm is obtained for SubPc, close to the value extracted from the SubPc-C_60_ system despite significantly increased recombination losses. This result illustrates the power of the photocurrent-ratio technique, even in a low-efficiency device with substantial recombination loss, the correct, intrinsic value of *L*_D_ is extracted. For the NPD dominant absorption region, an *η*_D_ (385 nm) = 47.1% is determined. Due to the large spectral overlap between NPD fluorescence and SubPc absorption, the dominant exciton harvesting mechanism within the NPD layer is Förster energy transfer to SubPc instead of exciton diffusion. In order to extract the *L*_D_ of NPD, a partner material should be chosen with reduced spectral overlap. Figure 4b shows the simulated *η*_EQE_ spectra for SubPc-NPD PHJ devices based on the parameters extracted. Unlike the SubPc-C_60_ case, simulated and experimental *η*_EQE_ spectra show broadband agreement as a function of SubPc thickness. This suggests that the exciton relaxation losses in SubPc-C_60_ devices originate only from excited states in C_60_.

Interestingly, the region (*λ* = 410–550 nm) where exciton relaxation losses are observed is consistent with the broad absorption peak of C_60_ bulk CT excitons^62^. These bulk CT excitons can be photoexcited directly and their absorption (*λ*_peak_ around 450 nm) has been observed to decrease drastically when diluting C_60_ in thin-film or solution^13,63^. This spectral agreement indicates that high energy bulk CT excitons do not uniformly relax to lowest energy Frenkel excitons in C_60_ despite their reasonably large energy difference (about 0.5 eV). For the C_60_ bulk CT excitons that fail to relax, they may still contribute to photocurrent through bulk-ionization (Fig. 4c), especially in devices with high built-in fields (see additional discussion in Supplementary Figure 5, Note 1). As such, both relaxation loss and bulk-ionization of bulk CT excitons must be considered in order to accurately simulate broadband *η*_EQE_ spectra of C_60_-based planar OPVs.

### Elucidating exciton quenching and dissociation pathways

In order to accurately probe intrinsic exciton diffusion in OPVs, the architecture of Fig. 1b was carefully designed to prevent exciton dissociation anywhere but at the D–A interface. As such, comparing its behavior to that of a conventional OPV allows additional exciton dissociation via electrode-organic interfaces and the built-in electric field to be quantified. For instance, removal of mCP between ITO and SubPc (10 nm) leads to a reduction in the SubPc exciton harvesting efficiency, permitting a quantification of quenching at the ITO interface. By inserting a wide gap interlayer to frustrate dissociation at the D–A interface, the photocurrent contribution from bulk-ionization is also quantified. Combining these results with an exciton diffusion model and importantly, accurate measurements of *L*_D_, the fraction of photogenerated excitons consumed by various quenching, dissociation, and recombination pathways can be determined, as shown in Supplementary Figure 6. For *λ* = 590 nm (mainly SubPc), the fraction of excitons dissociated at the D–A interface, quenched at the ITO-SubPc interface, and undergoing recombination within the donor layer are 73%, 21%, and 6%, respectively. For *λ* = 450 nm (mainly C_60_), 88% of C_60_ excitons relax to the lowest energy Frenkel states (47% dissociated at D–A interface and 41% recombining in the acceptor layer). Among the remaining 12% bulk CT excitons, 4% can contribute to photocurrent through bulk-ionization and 8% recombine prior to relaxation . These results suggest an important role for bulk-ionization via the built-in-field (especially for bulk CT excitons) in fullerene-based devices. This quantitative decoupling of photoconversion would not be accessible without the device-based *L*_D_ measurement reported here.

### Dark small molecules and other excitonic materials

**Fig. 5:**
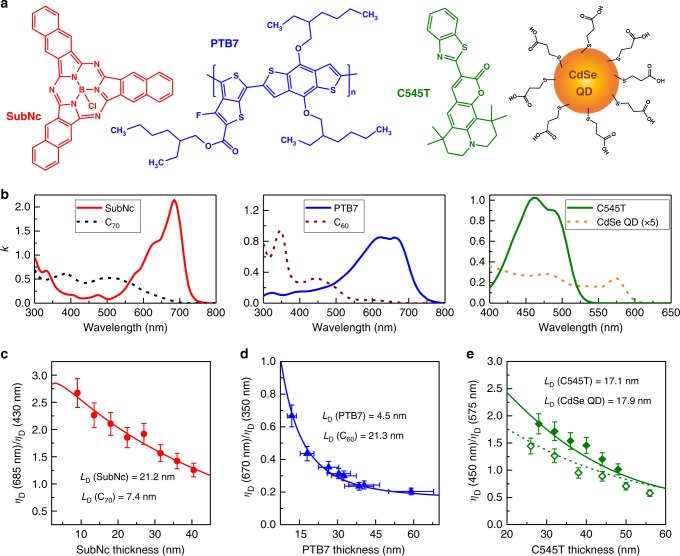
Measuring *L*_D_ of dark small molecules, polymers, and quantum dots. **a** Molecular structure of SubNc, PTB7, C545T and schematic of a CdSe quantum dot (QD) with 3-MPA ligands on the surface. **b** The extinction coefficients (k) of SubNc, C_70_, PTB7, C_60_, C545T, and CdSe QD thin films. The *k* of QDs is shown with a 5-fold magnification. **c** Diffusion efficiency ratio (*λ* = 685 nm to *λ* = 430 nm) as a function of SubNc layer thickness with the corresponding exciton diffusion length (*L*_D_) extracted from the fit (solid line) to the data. **d** Diffusion efficiency ratio (*λ* = 670 nm to *λ* = 350 nm) as a function of PTB7 layer thickness. **e** Diffusion efficiency ratio (*λ* = 450 nm to *λ* = 575 nm) as a function of C545T layer thickness and CdSe QD thickness (solid symbols: 66 nm QD layer; open symbols: 42 nm QD layer). The *L*_D_ values are extracted by fitting both sets of data simultaneously (solid line: fit for 66 nm QD devices; dash line: fit for 42 nm QD devices). Error bars of diffusion efficiency ratio represent run-to-run variation (standard deviation) for the devices tested. Error bars of PTB7 thickness were standard deviation of multiple polymer thin films fabricated with the same procedures

With the photocurrent-ratio method thoroughly vetted against conventional PL approaches for SubPc-C_60_, we investigate exciton transport in two dark small molecule materials, boron subnaphthalocyanine chloride (SubNc) and C_70_ (Fig. 5a). While these materials have been frequently utilized in high-efficiency OPVs, their intrinsic *L*_D_ has not been directly measured as they are inaccessible by PL-based techniques^7,12,58,64^. Similar to the SubPc-C_60_ system, we employ a SubNc-C_70_ PHJ device with varying donor thickness to extract the intrinsic *L*_D_ of both dark materials (structure: ITO/8.5 nm mCP/X ( = 9–40.5) nm SubNc/27 nm C_70_/11 nm BCP/1 nm MoO_x_/100 nm Al). Based on the extinction coefficients (k) of SubNc and C_70_ shown in Fig. 5b, the individual *η*_IQE_ of the donor and acceptor can be extracted from *λ* = 685 nm (SubNc absorption peak) and *λ* = 430 nm (primarily C_70_ absorption), respectively. By fitting the donor–acceptor *η*_D_ ratio (determined as previously described) as a function of SubNc thickness (Fig. 5c), we simultaneously extract an *L*_D_ of (21.2 ± 2.2) nm for SubNc and an *L*_D_ of (7.4 ± 0.8) nm for C_70_ (see Supplementary Figure 7 for *η*_EQE_, *η*_A_ and *η*_IQE_ spectra). The longer *L*_D_ of SubNc compared to SubPc (*L*_D_ = 16.7 nm) is consistent with previous observations based on the PL of dilute solid solutions^58^. The extracted *L*_D_ for C_70_ is significantly smaller than that of C_60_ (*L*_D_ = 18.5 nm). To better understand this difference, further work is needed to investigate differences in exciton lifetime and spin state between these fullerenes. Unlike the SubPc-C_60_ case, the simulated broadband *η*_EQE_ spectra of SubNc-C_70_ OPVs agree well with the experimental results, suggesting negligible exciton relaxation loss in C_70_. This result may reflect a reduced CT state character for excitons in C_70_.

To demonstrate the applicability of the photocurrent-ratio method in other excitonic systems, we also show two examples measuring exciton transport in solution processed PTB7 polymer films and inorganic CdSe quantum dot (QD) films. PTB7 is a high-performing polymer electron donor in OPVs, with demonstrated power efficiencies as high as 9.2% when combined with the fullerene acceptor PC_71_BM^65^. Here, we employ it as an electron-donor material paired with fullerene acceptor C_60_. Figure 5d shows the PTB7-C_60_ *η*_D_ ratio as a function of PTB7 thickness obtained from planar OPVs with a structure: ITO/2.5 nm HfO_2_/X ( = 12–59) nm PTB7/37 nm C_60_/10 nm BCP/1 nm MoO_x_/100 nm Al (see Supplementary Figure 8 for *η*_EQE_, *η*_A_ and *η*_IQE_ spectra). A thin layer of wide energy gap HfO_2_ is utilized as an exciton blocking layer at ITO/donor interface (deposited via atomic layer deposition). The donor *η*_IQE_ is extracted from *λ* = 670 nm (PTB7 absorption peak) while the acceptor *η*_IQE_ is extracted from *λ* = 350 nm (C_60_ Frenkel exciton absorption peak). As negligible exciton relaxation loss is observed at *λ* = 350 nm in the previous SubPc-C_60_ case (Fig. 4a), it is assumed that C_60_ has 100% exciton relaxation yield at this wavelength. Fitting the *η*_D_ ratio in Fig. 5d yields a short *L*_D_ of (4.5 ± 0.5) nm for PTB7 and an *L*_D_ of (21.3 ± 2.2) nm for C_60_. The C_60_ *L*_D_ is similar to the value extracted from the SubPc-C_60_ system (18.5 nm).

For CdSe QD thin films, the QDs are zincblende nanocrystals (size ~4 nm) stabilized with 3-mercaptopropionic acid (3-MPA) after treatment via solid-state ligand exchange^66^. The resulting thin films have a very low extinction coefficient (k = 0.05 at excitonic peak, *λ* = 575 nm), and are therefore paired with a wider energy gap small molecule 2,3,6,7-tetrahydro-1,1,7,7,-tetramethyl-1H,5 H,11H-10-(2-benzothiazolyl)quinolizino[9,9a,1gh]coumarin (C545T), which does not absorb at *λ* = 575 nm. Wide gap materials HfO_2_ and di-[4-(N,N-di-p-tolyl-amino)-phenyl]cyclohexane (TAPC) are utilized as exciton blocking layers for CdSe QDs and C545T, respectively. The CdSe QD-C545T planar cell has an inverted device architecture: ITO/2.5 nm HfO_2_/X (= 42, 66) nm CdSe QD/Y ( = 26–56) nm C545T/11 nm TAPC/10 nm MoO_x_/100 nm Al, where the CdSe QDs serves as the electron acceptor layer and photogenerated electrons are collected at ITO. It is again worth noting the ability of the photocurrent-ratio method in yielding intrinsic values of *L*_D_ even in devices with low efficiency. This allows active materials to be selected solely on the basis of realizing a minimal overlap in optical absorption. The QD film thickness is controlled by the number of cycles for the ligand exchange process^66^. Figure 5e shows the C545T-CdSe QD *η*_D_ ratio as a function of C545T thickness for two different QD film thicknesses (see Supplementary Figure 9 for broadband *η*_EQE_, *η*_A_ and *η*_IQE_ spectra). The donor *η*_IQE_ is extracted at *λ* = 450 nm (C545T absorption peak) while the acceptor *η*_IQE_ is extracted at *λ* = 575 nm (QD excitonic absorption peak). Results for devices with QD layer thickness of 42 nm and 66 nm are fitted simultaneously, yielding an *L*_D_ of (17.9 ± 3.6) nm for the CdSe QDs and an *L*_D_ of (17.1 ± 3.4) nm for C545T. It is worth noting that the *L*_D_ of C545T extracted here is consistent with separate PL-based measurements we have carried out (16.7 nm). If instead the *η*_D_ ratio is fit for each QD thickness, the 42 nm (66 nm) QD-C545T devices yield an *L*_D_ of 18.7 nm (15.5 nm) and 19.8 nm (16.8 nm) for the QDs and C545T, respectively. The *L*_D_ of CdSe QDs extracted here is smaller than that previously reported for CdSe core-shell counterparts (20 to 40 nm)^67,68^, likely reflecting that a wide gap shell is critical to frustrate exciton decay and enhance exciton transport for CdSe QDs.

## Discussion

We demonstrate a simple photocurrent-ratio method to measure the intrinsic *L*_D_ of excitonic semiconductors in bilayer photovoltaic cells. The impact of often unknown recombination losses is negated when taking a ratio of donor and acceptor *η*_IQE_. For the luminescent organic pairing SubPc and C_60_, their *L*_D_ extracted using this method are in excellent agreement with those extracted from conventional PL-quenching measurements, a direct result of the ability to remove the role of unknown geminate recombination losses. With knowledge of the intrinsic values of *L*_D_, we are able to investigate the potential for relaxation losses, which are typically overlooked in device-based studies of *L*_D_. Indeed, bulk CT excitons generated in C_60_ have non-unity relaxation yield to Frenkel states and can generate free carriers directly through bulk-ionization. The generality of this photocurrent-ratio method is further demonstrated by extracting *L*_D_ for the dark small molecule SubNc, C_70_, the high-performance polymer PTB7, as well as for CdSe quantum dot thin films. Thus, we demonstrate a straightforward measurement for the extraction of *L*_D_ that is equally applicable to both luminescent and dark excitonic materials capable of being integrated into a bilayer photovoltaic cell.

## Methods

### Chemicals

For this study, SubPc (99%), mCP (99%), TPBi (99%), C545T (99%), TAPC (99%), and PTB7 (Mw > 50,000) were obtained from Lumtec Inc., C_60_ (99%) and C_70_ (99%) were obtained from MER Corporation, MoO_3_ (99%) and BCP (98%) were obtained from Alfa Aesar, chloroform (99%), 3-mercaptopropionic acid (99%), octane (98%), oleic acid (tech. grade 90%) acetonitrile (99.5%), and methyl acetate (anhydrous) were obtained from Sigma–Aldrich, selenium dioxide (99.8%) and 1-octadecene (90%) were obtained from Acros Organics. All materials above were used as received. The NPD (99%) was synthesized by the Dow Chemical Company and purified once by temperature-gradient sublimation^69^. Cadmium myristate was prepared on a multi-gram scale according to the method reported by Chen et al.^70^.

### Synthesis of zincblende CdSe QDs

Zincblende CdSe QDs with oleic acid capping ligands were synthesized as previously reported^66^, based off the original synthesis by Chen and et al.^70^. Briefly, 1-octadecene (63 mL), cadmium myristate (570 mg) and selenium dioxide (110 mg) were added to a 3-neck round bottom flask and degassed at 75 °C for 1 h. After degassing, the reaction mixture was heated to 240 °C, at which point 1 mL of oleic acid was added dropwise to the solution once a red color was achieved. The reaction was allowed to proceed for 15 min and then cooled to room temperature for purification by centrifugation in methyl acetate. The resulting precipitate was dispersed in octane and filtered using a 0.2 um PTFE syringe filter prior to film fabrication. Resulting solution concentration was ~25 g L^−1^.

### Thin-film and device preparation

Organic photovoltaic cells were fabricated using indium-tin-oxide (ITO)-coated glass substrates with a sheet resistance of 8–12 Ω per □. Substrates were cleaned in tergitol solution and in organic solvents and treated in UV-Ozone ambient for 10 min prior to thin-film deposition. All polymer and QD films were spin-coated onto an HfO_2_-coated ITO substrate. The HfO_2_ was deposited via atomic layer deposition at 200 °C. The polymer PTB7 was first dispersed in chloroform and then spin-coated at 3000 rpm for 45 s. The obtained samples were annealed in N_2_ at 140 °C for 10 min. The polymer film thickness was controlled via the concentration of the PTB7 solution (2–10 g L^−1^). Spin-coating and buildup of QD film thickness were realized using a solid-state ligand exchange procedure. CdSe QDs dispersed in octane were spin-coated at 1200 rpm for 30 s and then 6000 rpm for 60 s. The latter process is found to improve film uniformity and reproducibility. Next, 3-MPA (1% v/v) in acetonitrile was first deposited onto the film and allowed to sit for 10 s to facilitate the ligand exchange. The sample was then spun again at 1200 rpm for 30 s. Finally, the film was rinsed with pure acetonitrile by spinning at 6000 rpm for 60 s. This process was repeated 3–4 times until the desired QD film thickness was achieved. All other layers were deposited at room temperature by high vacuum thermal evaporation at a pressure of less than 8 × 10^–7^ Torr. The active area of the obtained device is 0.785 mm^2^, defined by the cathode area.

### Optoelectronic characterization

Photoluminescence quenching data were recorded using a Photon Technology International QuantaMaster Fluorometer. All photoluminescence measurements were performed under N_2_ purge. Photoluminescence quenching measurements were made at an incident angle of 70° to the substrate normal. External quantum efficiency measurements were collected by measuring the photocurrent under monochromatic light illumination using a 300 W Oriel Xe lamp, a monochromator, an optical chopper wheel, and an SR810 lock-in amplifier. Error bars for *L*_D_ represent a 95% confidence interval extracted from fitting parameters. All film thicknesses, reflectivity spectra were measured with a J. A. Woollam spectroscopic ellipsometer. The device reflectivity measurements were made at an incident angle of 15° to the substrate normal. Film thicknesses were fit using a Cauchy model. For simulations of device absorption efficiency optical constants of organic and QD thin films were extracted by fitting transmission (at normal incidence) and reflection (incident angle: 15°) simultaneously with a transfer matrix formalism (provided in Supplementary Figure 10).

## Supplementary information

Supplementary Information

## Data Availability

The data presented within this manuscript are available from the corresponding author upon reasonable request.

## References

[1] Ye Z (2014). Probing excitonic dark states in single-layer tungsten disulphide. Nature.

[2] Ye Y (2015). Monolayer excitonic laser. Nat. Photonics.

[3] Blancon JC (2017). Extremely efficient internal exciton dissociation through edge states in layered 2d perovskites. Science.

[4] Zhang Q, Chu L, Zhou F, Ji W, Eda G (2018). Excitonic properties of chemically synthesized 2D organic–inorganic hybrid perovskite nanosheets. Adv. Mater..

[5] Milichko VA (2017). Van der waals metal-organic framework as an excitonic material for advanced photonics. Adv. Mater..

[6] Najafov H, Lee B, Zhou Q, Feldman LC, Podzorov V (2010). Observation of long-range exciton diffusion in highly ordered organic semiconductors. Nat. Mater..

[7] Cnops K (2014). 8.4% efficient fullerene-free organic solar cells exploiting long-range exciton energy transfer. Nat. Commun..

[8] Shirasaki Y, Supran GJ, Bawendi MG, Bulović V (2013). Emergence of colloidal quantum-dot light-emitting technologies. Nat. Photonics.

[9] Sun Y (2006). Management of singlet and triplet excitons for efficient white organic light-emitting devices. Nature.

[10] Coburn C, Lee J, Forrest SR (2016). Charge balance and exciton confinement in phosphorescent organic light emitting diodes. Adv. Opt. Mater..

[11] Zhang S, Qin Y, Zhu J, Hou J (2018). Over 14% efficiency in polymer solar cells enabled by a chlorinated polymer donor. Adv. Mater..

[12] Che X, Li Y, Qu Y, Forrest SR (2018). High fabrication yield organic tandem photovoltaics combining vacuum- and solution-processed subcells with 15% efficiency. Nat. Energy.

[13] Che X, Xiao X, Zimmerman JD, Fan D, Forrest SR (2014). High-efficiency, vacuum‐deposited, small-molecule organic tandem and triple-junction photovoltaic cells. Adv. Energy Mater..

[14] Zhao W (2017). Molecular optimization enables over 13% efficiency in organic solar cells. J. Am. Chem. Soc..

[15] Lin Y (2017). Mapping polymer donors toward high-efficiency fullerene free organic solar cells. Adv. Mater..

[16] Deotare PB (2015). Nanoscale transport of charge-transfer states in organic donor-acceptor blends. Nat. Mater..

[17] High AA, Novitskaya EE, Butov LV, Hanson M, Gossard AC (2008). Control of exciton fluxes in an excitonic integrated circuit. Science.

[18] Unuchek D (2018). Room-temperature electrical control of exciton flux in a van der waals heterostructure. Nature.

[19] Lin JD (2014). Systematic study of exciton diffusion length in organic semiconductors by six experimental methods. Mater. Horiz..

[20] Mikhnenko OV, Blom PW, Nguyen TQ (2015). Exciton diffusion in organic semiconductors. Energy Environ. Sci..

[21] Menke SM, Holmes RJ (2014). Exciton diffusion in organic photovoltaic cells. Energy Environ. Sci..

[22] Hedley GJ, Ruseckas A, Samuel ID (2016). Light harvesting for organic photovoltaics. Chem. Rev..

[23] Scharber MC (2006). Design rules for donors in bulk-heterojunction solar cells—towards 10% energy-conversion efficiency. Adv. Mater..

[24] Kippelen B, Brédas JL (2009). Organic photovoltaics. Energy Environ. Sci..

[25] Park SH (2009). Bulk heterojunction solar cells with internal quantum efficiency approaching 100%. Nat. Photonics.

[26] Mayer AC, Scully SR, Hardin BE, Rowell MW, McGehee MD (2007). Polymer-based solar cells. Mater. Today.

[27] Halls JJ, Pichler K, Friend RH, Moratti S, Holmes A (1996). Exciton diffusion and dissociation in a poly (p-phenylenevinylene)/C_60_ heterojunction photovoltaic cell. Appl. Phys. Lett..

[28] Tamai Y, Ohkita H, Benten H, Ito S (2015). Exciton diffusion in conjugated polymers: from fundamental understanding to improvement in photovoltaic conversion efficiency. J. Phys. Chem. Lett..

[29] Westenhoff S, Howard IA, Friend RH (2008). Probing the morphology and energy landscape of blends of conjugated polymers with sub-10 nm resolution. Phys. Rev. Lett..

[30] Penwell SB, Ginsberg LDS, Noriega R, Ginsberg NS (2017). Resolving ultrafast exciton migration in organic solids at the nanoscale. Nat. Mater..

[31] Kose ME (2009). Exciton migration in conjugated dendrimers: ajoint experimental and theoretical study. Chemphyschem.

[32] Luhman WA, Holmes RJ (2011). Investigation of energy transfer in organic photovoltaic cells and impact on exciton diffusion length measurements. Adv. Funct. Mater..

[33] Mikhnenko OV (2012). Exciton diffusion length in narrow bandgap polymers. Energy Environ. Sci..

[34] Thompson NJ (2014). Energy harvesting of non-emissive triplet excitons in tetracene by emissive PbS nanocrystals. Nat. Mater..

[35] Congreve DN (2013). External quantum efficiency above 100% in a singlet-exciton-fission–based organic photovoltaic cell. Science.

[36] Efros AL (1996). Band-edge exciton in quantum dots of semiconductors with a degenerate valence band: dark and bright exciton states. Phys. Rev. B.

[37] Zou Y, Holst J, Zhang Y, Holmes RJ (2014). 7.9% efficient vapor-deposited organic photovoltaic cells based on a simple bulk heterojunction. J. Mater. Chem. A.

[38] Bergemann K. J., Liu X., Panda A. & Forrest S. R. Singlets lead to photogeneration in C_60_-based organic heterojunctions. *Phys. Rev. B* **92**, 035408 (2015).

[39] Peumans P, Yakimov A, Forrest SR (2003). Small molecular weight organic thin-film photodetectors and solar cells. J. Appl. Phys..

[40] Luhman WA, Holmes RJ (2009). Enhanced exciton diffusion in an organic photovoltaic cell by energy transfer using a phosphorescent sensitizer. Appl. Phys. Lett..

[41] Pettersson LAA, Roman LS, Inganas O (1999). Modeling photocurrent action spectra of photovoltaic devices based on organic thin films. J. Appl. Phys..

[42] Terao Y, Sasabe H, Adachi C (2007). Correlation of hole mobility, exciton diffusion length, and solar cell characteristics in phthalocyanine/fullerene organic solar cells. Appl. Phys. Lett..

[43] Kim I (2009). Efficient organic solar cells based on planar metallophthalocyanines. Chem. Mater..

[44] Guide M (2014). Effect of copper metalation of tetrabenzoporphyrin donor material on organic solar cell performance. J. Mater. Chem. A.

[45] Siegmund B (2017). Exciton diffusion length and charge extraction yield in organic bilayer solar cells. Adv. Mater..

[46] Mullenbach TK, Curtin IJ, Zhang T, Holmes RJ (2017). Probing dark exciton diffusion using photovoltage. Nat. Commun..

[47] Zhang T, Holmes RJ (2017). Photovoltage as a quantitative probe of carrier generation and recombination in organic photovoltaic cells. J. Mater. Chem. C.

[48] Vandewal K (2014). Efficient charge generation by relaxed charge-transfer states at organic interfaces. Nat. Mater..

[49] Kurpiers J (2018). Probing the pathways of free charge generation in organic bulk heterojunction solar cells. Nat. Commun..

[50] Curtin I. J. & Holmes R. J. Decoupling photocurrent loss mechanisms in photovoltaic cells using complementary measurements of exciton diffusion. *Adv. Energy Mater*. **8**, 1702339 (2018). 10.1002/aenm.201702339.

[51] Credgington D, Liu SW, Nelson J, Durrant JR (2014). In situ measurement of energy level shifts and recombination rates in subphthalocyanine/C_60_ bilayer solar cells. J. Phys. Chem. C.

[52] Pandey R, Holmes RJ (2012). Characterizing the charge collection efficiency in bulk heterojunction organic photovoltaic cells. Appl. Phys. Lett..

[53] Zou Y, Holmes RJ (2013). Influence of a moox interlayer on the open-circuit voltage in organic photovoltaic cells. Appl. Phys. Lett..

[54] Yi Y (2009). The interface state assisted charge transport at the MoO(3)/metal interface. J. Chem. Phys..

[55] Zou Y, Holmes RJ (2015). The role of exciton ionization processes in bulk heterojunction organic photovoltaic cells. Adv. Energy Mater..

[56] Chandran HT (2017). Direct free carrier photogeneration in single layer and stacked organic photovoltaic devices. Adv. Mater..

[57] Proctor CM, Kuik M, Nguyen TQ (2013). Charge carrier recombination in organic solar cells. Prog. Polym. Sci..

[58] Menke SM, Holmes RJ (2015). Energy-cascade organic photovoltaic devices incorporating a host-guest architecture. ACS Appl. Mater. Interfaces.

[59] Lunt RR, Giebink NC, Belak AA, Benziger JB, Forrest SR (2009). Exciton diffusion lengths of organic semiconductor thin films measured by spectrally resolved photoluminescence quenching. J. Appl. Phys..

[60] Menke SM, Luhman WA, Holmes RJ (2013). Tailored exciton diffusion in organic photovoltaic cells for enhanced power conversion efficiency. Nat. Mater..

[61] Pandey R, Gunawan AA, Mkhoyan KA, Holmes RJ (2012). Efficient organic photovoltaic cells based on nanocrystalline mixtures of boron subphthalocyanine chloride and C_60_. Adv. Funct. Mater..

[62] Hahn T (2016). Role of intrinsic photogeneration in single layer and bilayer solar cells with c60 and pcbm. J. Phys. Chem. C.

[63] Ishibashi Y, Arinishi M, Katayama T, Miyasaka H, Asahi T (2018). Femtosecond excited-state dynamics of fullerene-C_60_ nanoparticles in water. Phys. Chem. Chem. Phys..

[64] Li Y (2018). Near-infrared ternary tandem solar cells. Adv. Mater..

[65] He Z (2012). Enhanced power-conversion efficiency in polymer solar cells using an inverted device structure. Nat. Photonics.

[66] Dement DB, Puri M, Ferry VE (2018). Determining the complex refractive index of neat CdSe/CdS quantum dot films. J. Phys. Chem. C.

[67] Akselrod GM (2014). Subdiffusive exciton transport in quantum dot solids. Nano. Lett..

[68] Lee EMY, Tisdale WA (2015). Determination of exciton diffusion length by transient photoluminescence quenching and its application to quantum dot films. J. Phys. Chem. C.

[69] Curtin IJ, Blaylock DW, Holmes RJ (2016). Role of impurities in determining the exciton diffusion length in organic semiconductors. Appl. Phys. Lett..

[70] Chen O (2008). Synthesis of metal–selenide nanocrystals using selenium dioxide as the selenium precursor. Angew. Chem. Int. Ed..

