# Supplementary material for: Intrinsic measurements of exciton transport in photovoltaic cells
Source: Nat Commun. 2019 Mar 11;10:1156. doi: 10.1038/s41467-019-09062-8 (PMC6411876; doi:10.1038/s41467-019-09062-8)
Supplement: Supplementary file 1 — Supplementary Information [file 41467_2019_9062_MOESM1_ESM.pdf]

Supplementary Information

Intrinsic Measurements of Exciton Transport in Photovoltaic Cells

Zhang et al.

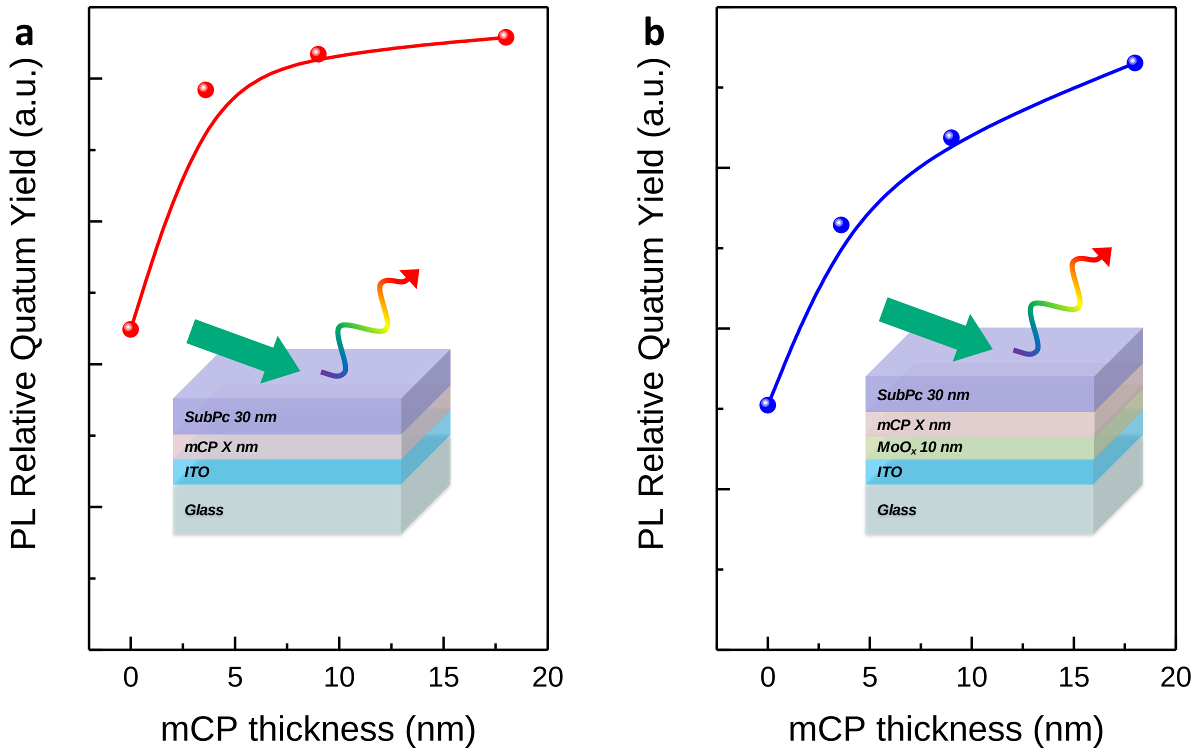

Supplementary Figure 1 | Preventing exciton loss at SubPc-ITO and SubPc-MoO_x interface. Photoluminescence (PL) relative quantum yield of SubPc films as a function of mCP thickness between SubPc and ITO/MoO_x. The PL relative quantum yield calculated as the ratio of integrated PL to the number of generated excitons. Exciton generation is simulated by a transfer matrix model[1]. SubPc films are all pumped with λ = 500 nm light. The incident angle is 70° for all incident light. An increase in PL relative quantum yield with mCP (exciton blocking) thickness suggests exciton loss at SubPc-ITO and SubPc-MoO_x interfaces.

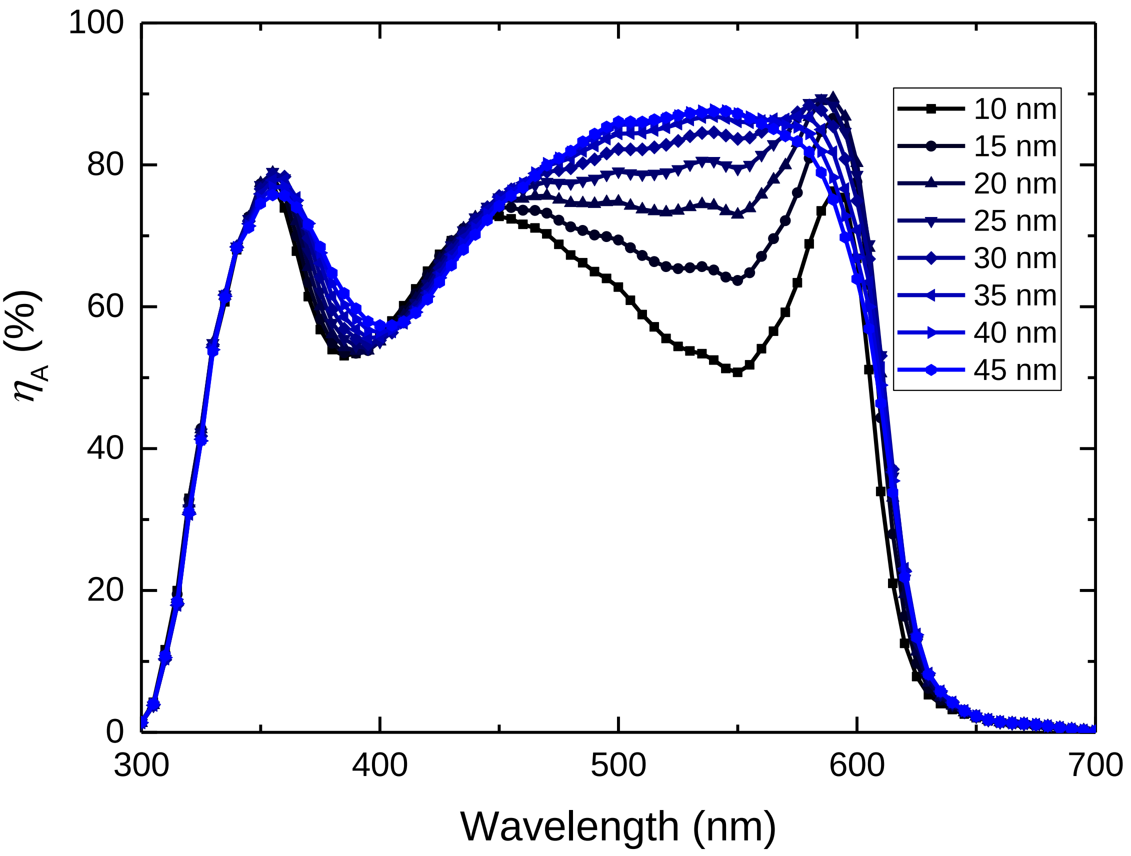

Supplementary Figure 2 | Absorption efficiency spectra of SubPc-C_{60} planar OPVs. The absorption efficiency (η_A) spectra of devices in Fig. 1b are calculated using transfer matrix model. The plotted η_A is the total active layer absorption efficiency, including the absorption of both SubPc and C_{60}.

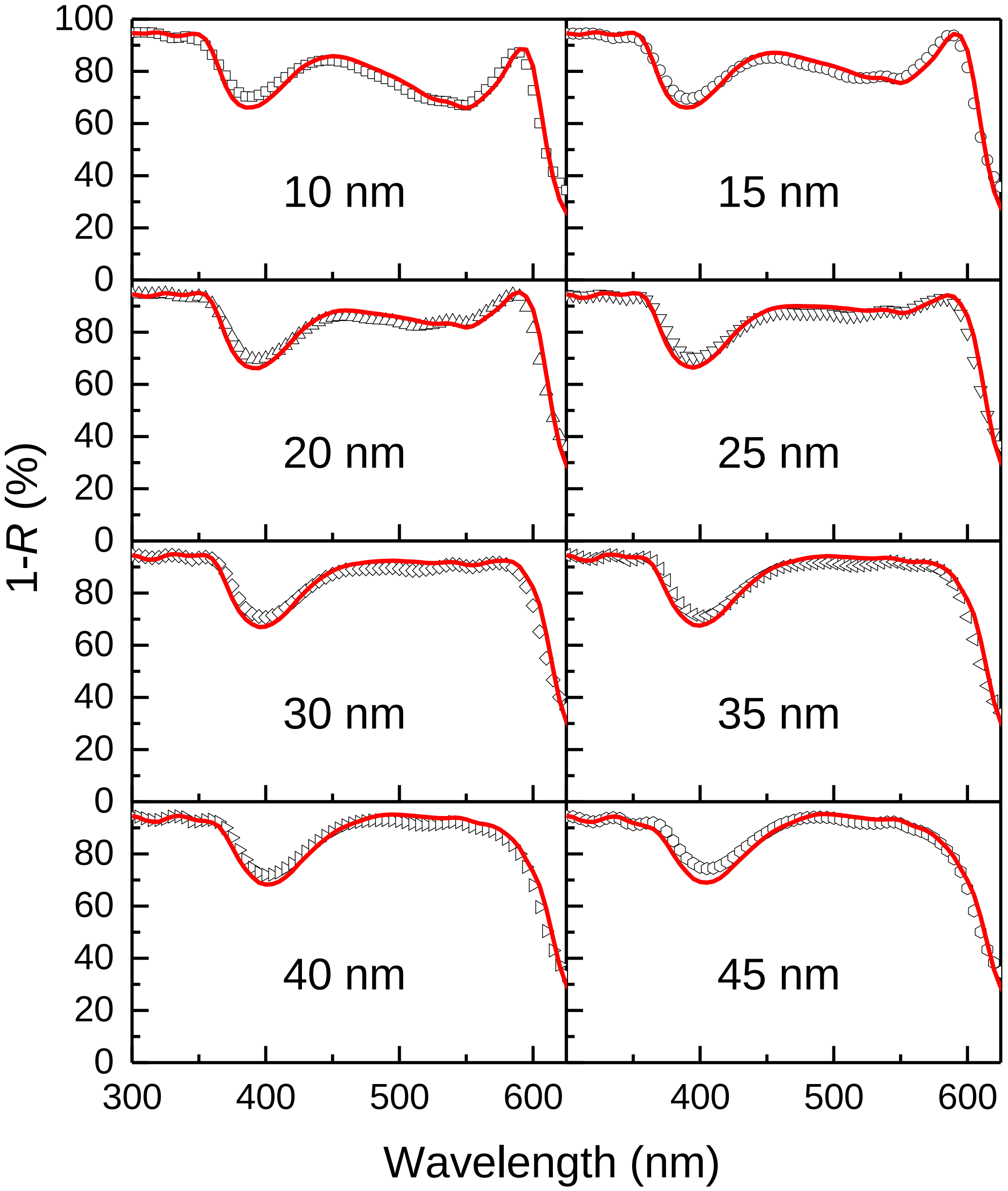

Supplementary Figure 3 | Experimental and simulated reflectivity of SubPc-C_{60} planar OPVs. The reflectivity (*R*) of devices in Fig. 1b is measured off the Al cathode through the ITO/organic layers. The reflectivity measurements were made at an incident angle of 15° to the substrate normal. The experimental results are shown in symbols and the simulation is shown as a red solid line.

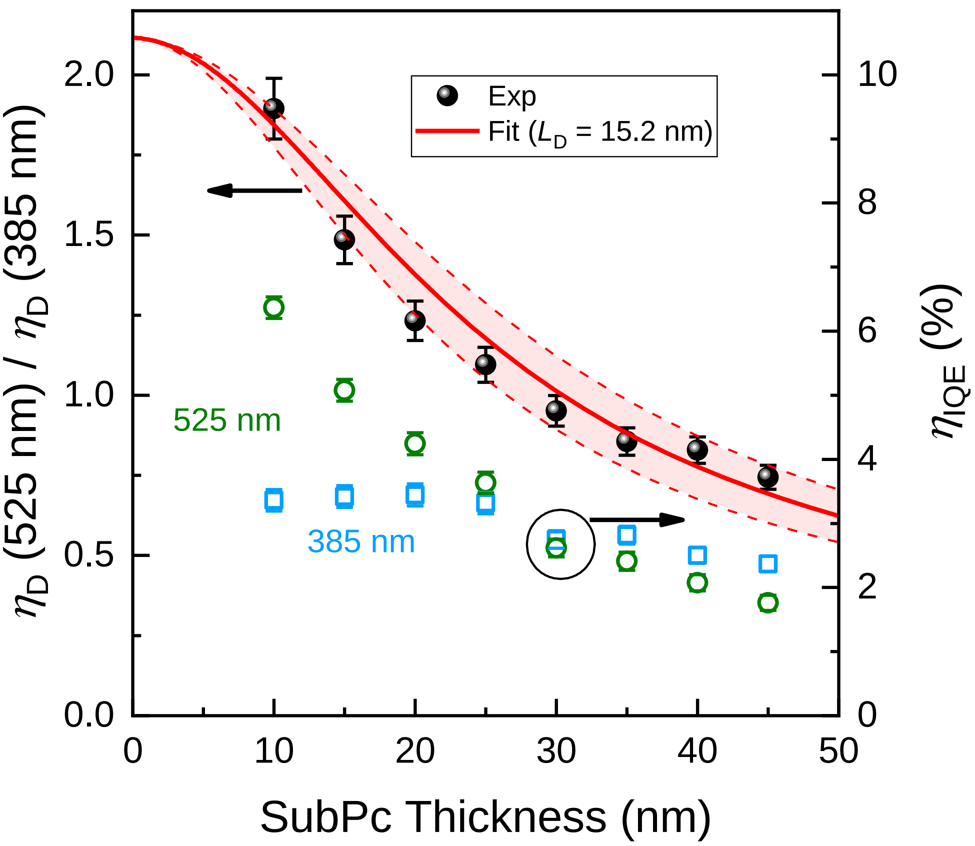

Supplementary Figure 4 | Extracting exciton diffusion length from of SubPc-NPD planar OPVs. Diffusion efficiency ratios (λ = 525 nm to λ = 385 nm) as a function of SubPc thickness for SubPc-NPD planar OPVs. The red solid line is the best fit of the data, corresponding to a SubPc L_D of 15.2 nm. The internal quantum efficiency at wavelengths of λ = 385 nm and λ = 575 nm is shown on the right axis. Error bars represent the standard deviation of measured devices.

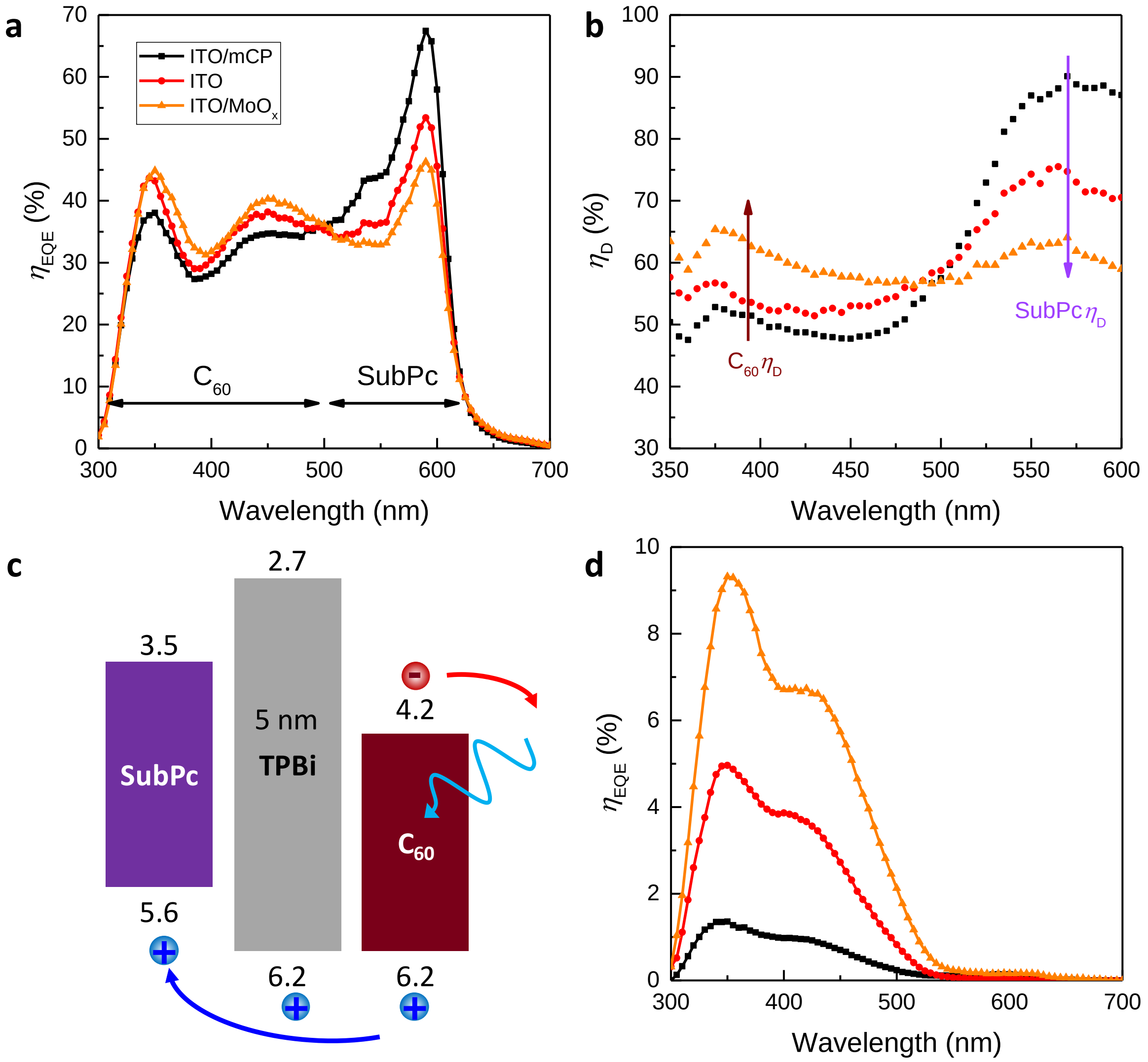

Supplementary Figure 5 | Impact of anode buffer layer on exciton harvesting. a, External quantum efficiency spectra for SubPc-C_{60} OPVs with structure: ITO/7 nm mCP or no buffer layer or 10 nm MoO_x/10 nm SubPc/35 nm C_{60}/10 nm BCP/1 nm MoOx/ 100 nm Al. Horizontal arrows denote the spectral regions of dominant absorption for SubPc and C_{60}. **b,** Diffusion efficiency spectra determined from the external quantum efficiency in (a) by assuming unity charge separation efficiency. The purple and brown arrows show the impact of anode buffer layers on exciton harvesting in SubPc and C_{60}, respectively. **c,** Schematic of photocurrent generation in a SubPc-TPBi-C_{60} planar device made by inserting 5-nm-thick TPBi interlayer at D-A interface of devices in (a). **d,** The external quantum efficiency spectra of SubPc-TPBi-C_{60} planar devices.

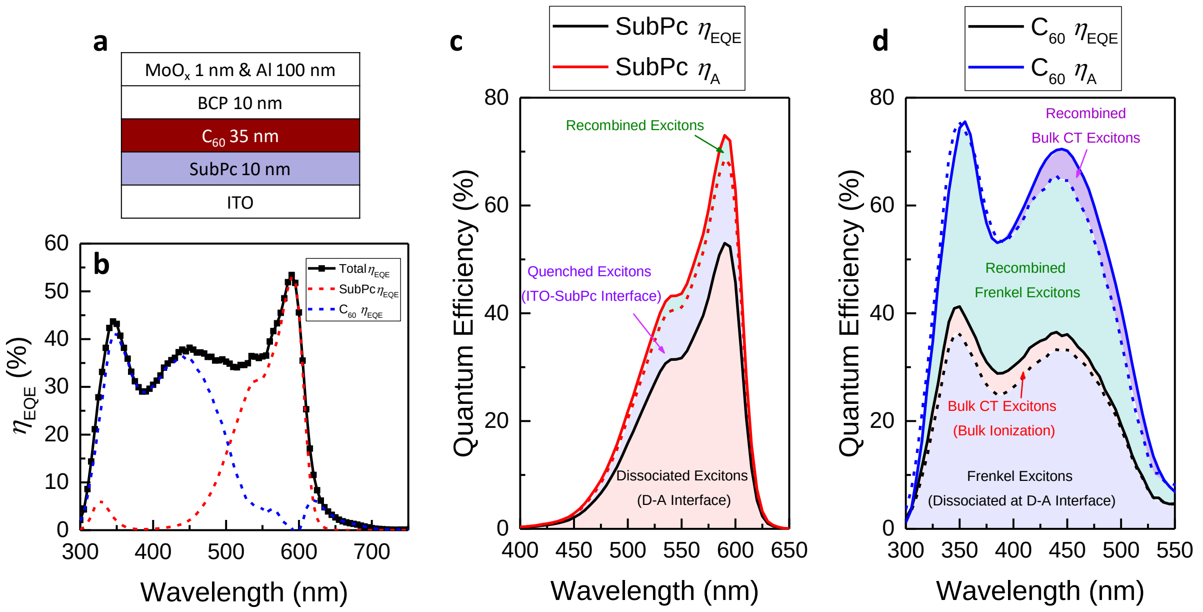

Supplementary Figure 6 | Decoupling exciton quenching and dissociation in an OPV. a, Device architecture of SubPc-C_{60} planar OPV used to assess losses related to quenching at the ITO electrode. **b,** External quantum efficiency spectra for the SubPc-C_{60} OPV in (a). The donor and acceptor contributions are shown as dashed lines. **c,** Decoupling for photogeneration in SubPc. Red solid line is the SubPc absorption efficiency spectrum simulated by transfer matrix model. Shaded regions indicate the magnitude of each exciton relaxation pathway. **d,** Decoupling exciton quenching and dissociation for photogeneration in C_{60}. Blue solid line is the C_{60} absorption efficiency simulated using an optical transfer matrix model.

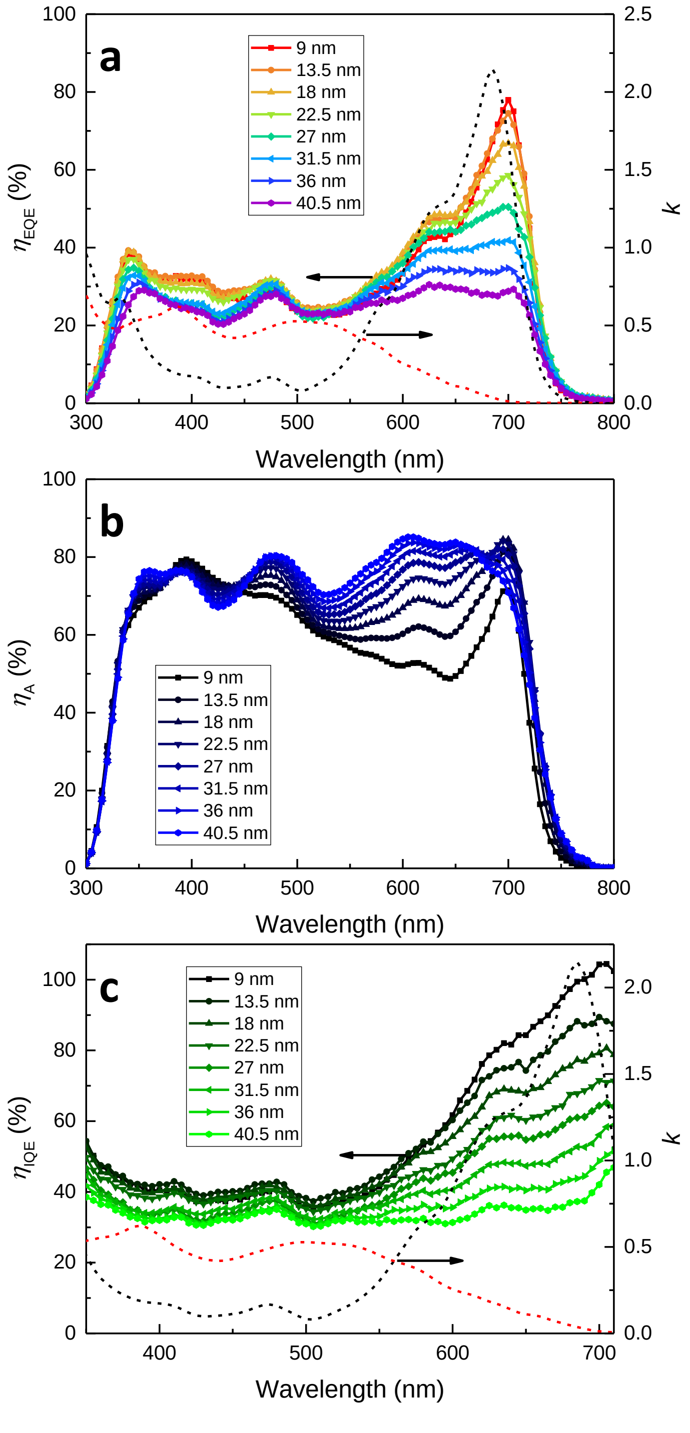

Supplementary Figure 7 | Quantum efficiency spectra for SubNc-C_{70} planar OPVs. a, The η_{EQE} spectra measured at short-circuit as a function of SubNc layer thickness. The devices have the structure: ITO/8.5 nm mCP/X (=9-40.5) nm SubNc/27 nm C_{70}/11 nm BCP/1 nm MoO_x/100 nm Al. **b,** The η_A spectra calculated using a transfer matrix model. **c,** The η_{IQE} spectra calculated by dividing the η_{EQE} spectra in **a** by the η_A spectra in **b**. The extinction coefficients (*k*) of SubNc (black dash line) and C_{70} (red dash line) are also shown.

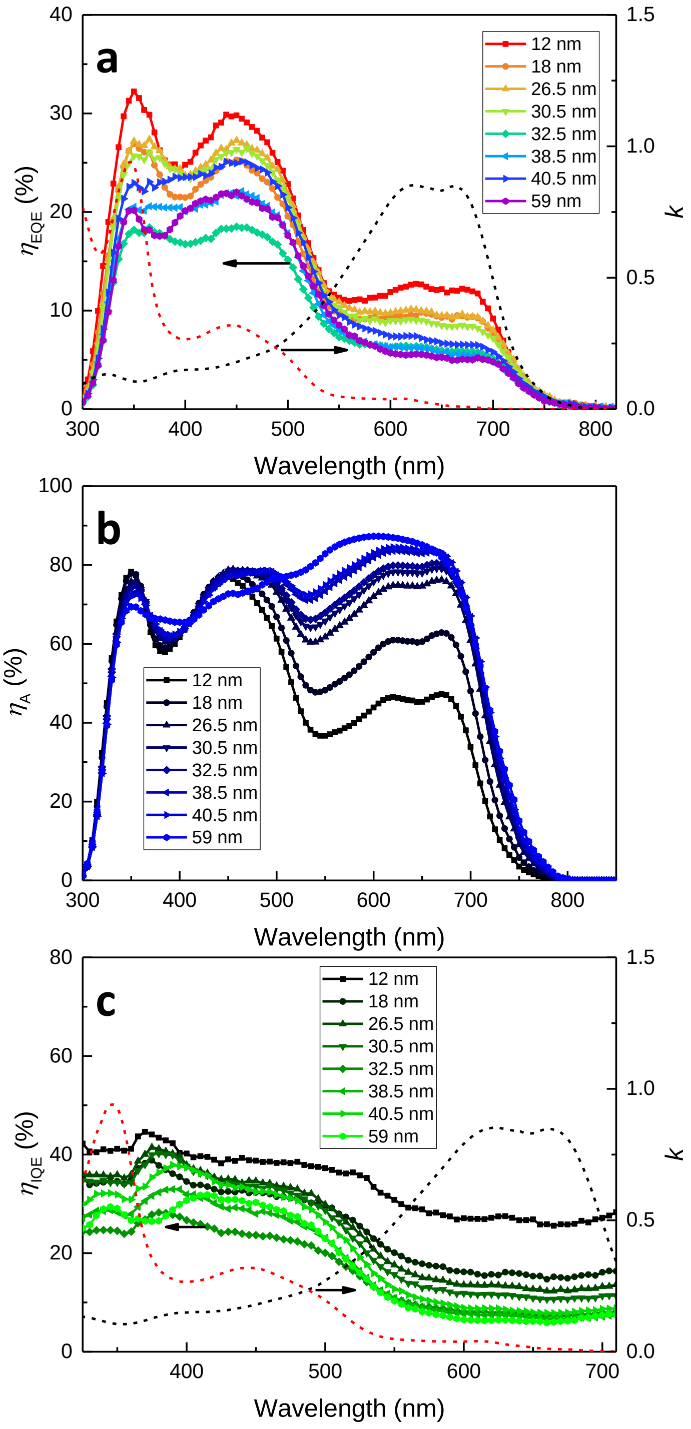

Supplementary Figure 8 | Quantum efficiency spectra for PTB7-C_{60} planar OPVs. a, The η_{EQE} spectra measured at short-circuit as a function of PTB7 polymer layer thickness. The devices have the structure: ITO/2.5 nm HfO_2/X (=12-59) nm PTB7/37 nm C_{60}/10 nm BCP/1 nm MoO_x/100 nm Al. **b,** The η_A spectra calculated using a transfer matrix model. **c,** The η_{IQE} spectra calculated by dividing the η_{EQE} spectra in **a** by the η_A spectra in **b**. The extinction coefficients (*k*) of PTB7 (black dash line) and C_{60} (red dash line) are also shown.

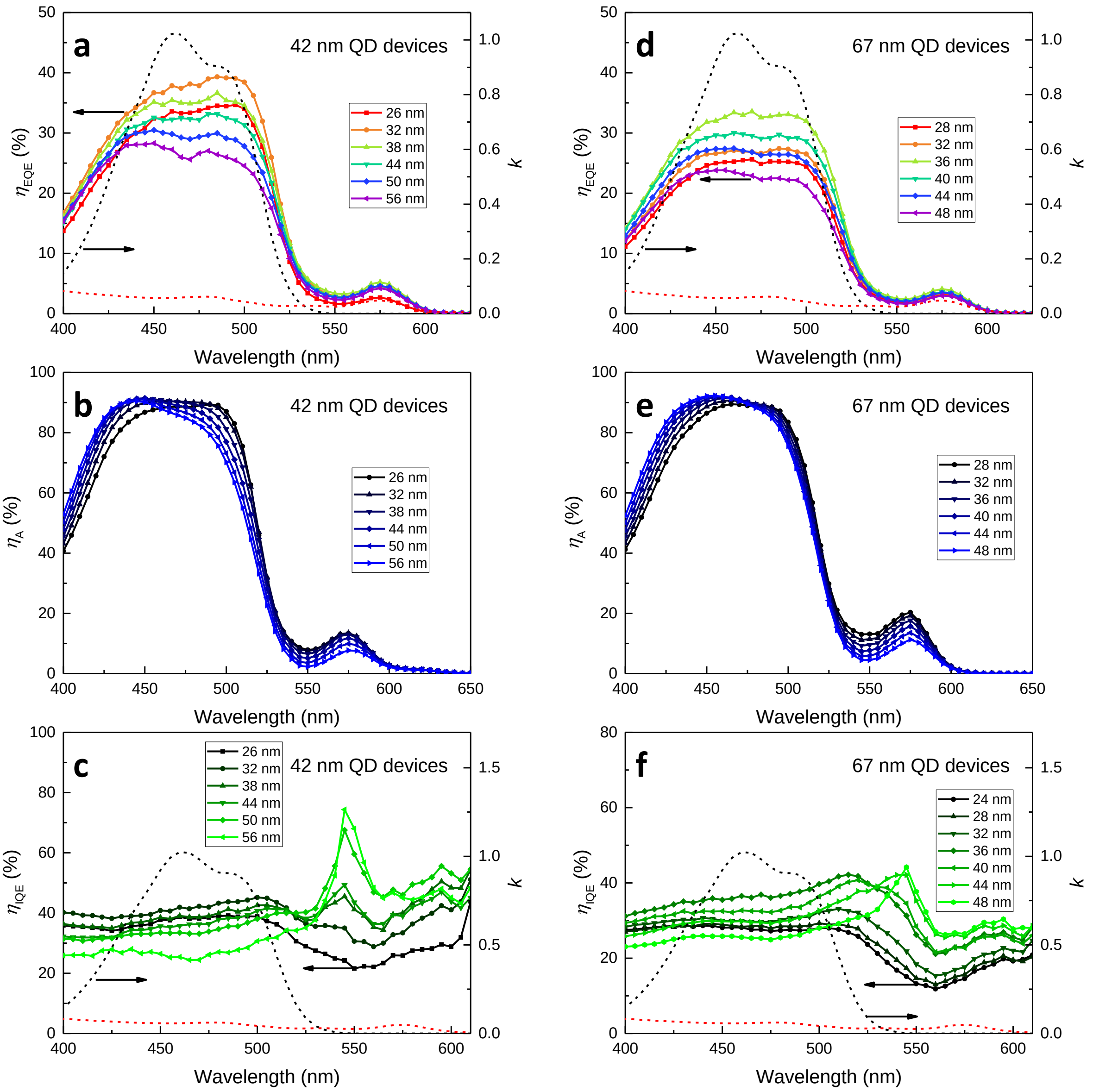

Supplementary Figure 9 | Quantum efficiency spectra for CdSe QD-C545T planar photovoltaic cells. a, The η_{EQE} spectra measured at short-circuit as a function of C545T layer thickness. The devices have the structure: ITO/2.5 nm HfO_2/42 nm CdSe QD/Y (=26-56) nm C545T/11 nm TAPC/10 nm MoO_x/100 nm Al. **b,** The η_A spectra of devices in **a** calculated using a transfer matrix model. **c,** The η_{IQE} spectra calculated by dividing the η_{EQE} spectra in **a** by the η_A spectra in **b**. **d,** The η_{EQE} spectra of devices as a function of C545T layer thickness. Device structure: ITO/2.5 nm HfO_2/67 nm CdSe QD/Y (=24-48) nm C545T/11 nm TAPC/10 nm MoO_x/100 nm Al. **e,** The η_A spectra of devices in **d**. **f,** The η_{IQE} spectra calculated by dividing the η_{EQE} spectra in **d** by the η_A spectra in **e**. The extinction coefficients (k) of C545T (black dash line) and CdSe QD (red dash line) are also shown.

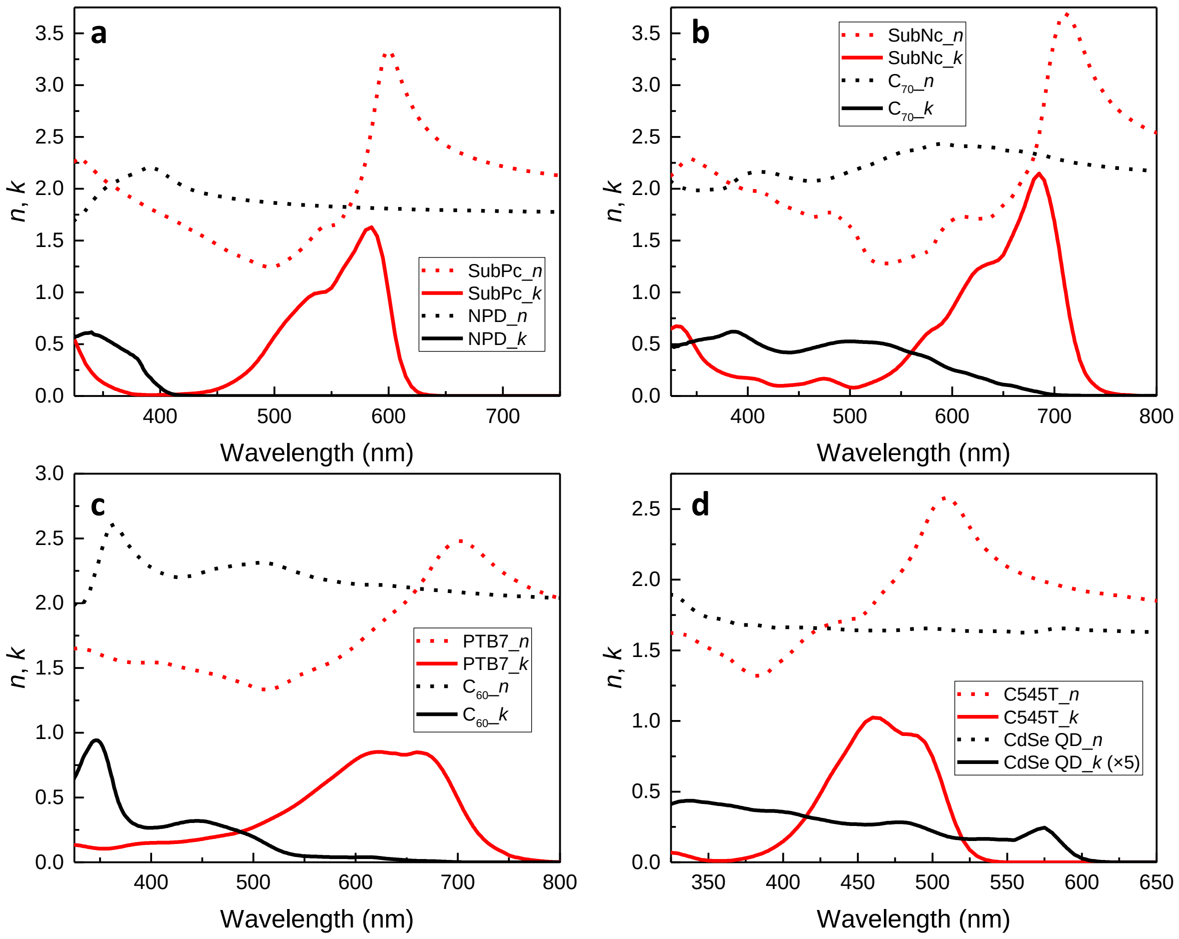

Supplementary Figure 10 | Optical constants of photoactive materials for L_D measurements. The refractive index *n* (dash line) and the extinction coefficient *k* (solid line) of (**a**) SubPc, NPD (**b**) SubNc, C_{70} (**a**) PTB7, C_{60} (**d**) C545T, CdSe quantum dots (QDs). The *k* of CdSe QDs is shown with a 5-fold magnification.

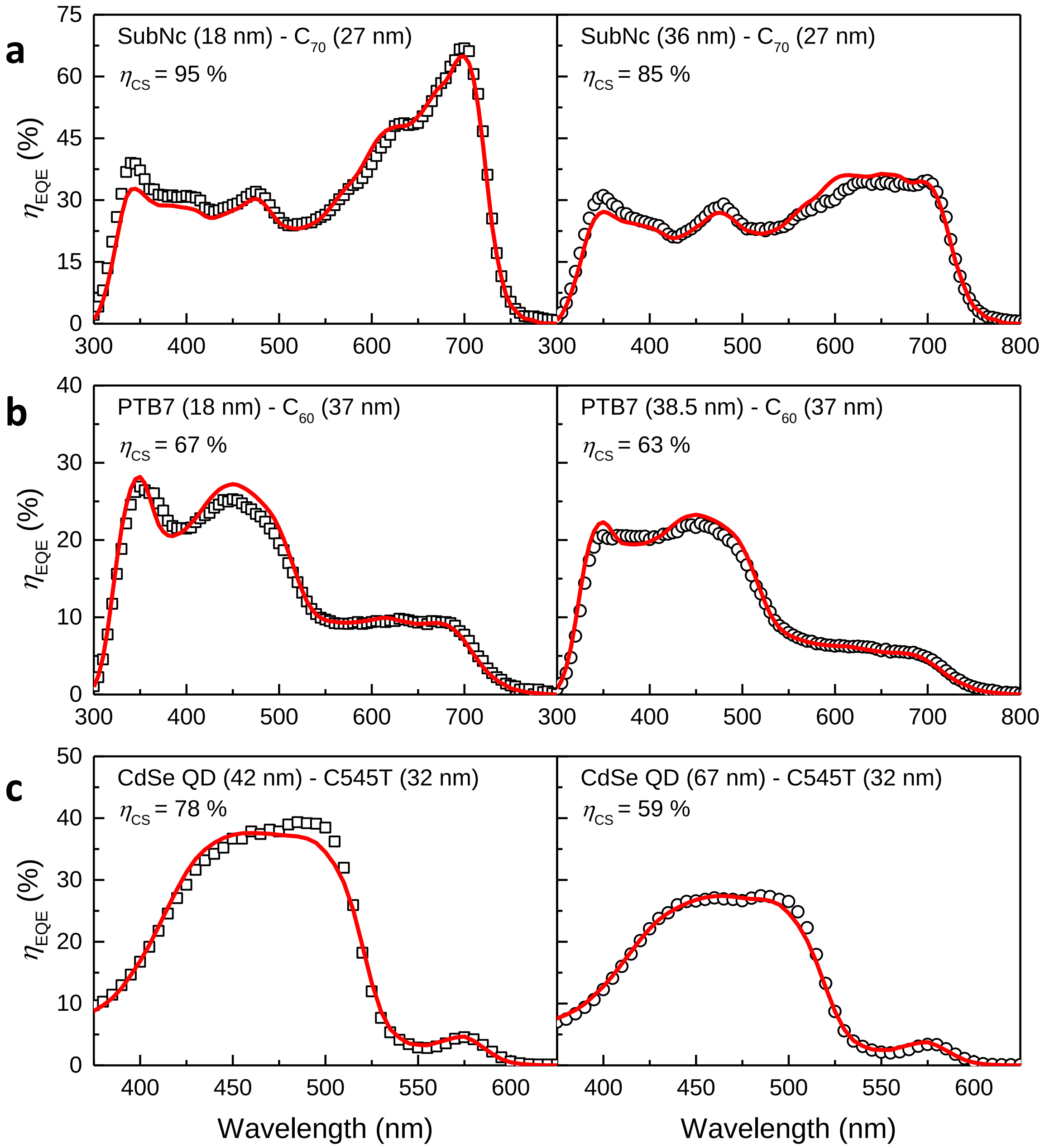

Supplementary Figure 11 | Simulation of external quantum efficiency for extraction of charge separation efficiency. a, The η_{EQE} spectra for SubNc-C_{70} planar OPVs in Supplementary Figure 7 as a function of SubNc thickness (18 and 36 nm). Experimental results are shown in symbols while solid lines are simulated spectra (simulated using L_D values shown in Fig. 5). **b,** The η_{EQE} spectra for PTB7-C_{60} planar OPVs in Supplementary Figure 8 versus PTB7 thickness (18 and 38.5 nm). The simulated η_{EQE} spectra show slight overestimation compared to experimental results for wavelength ~450 nm, similar to the SubPc-C_{60} case with non-unity exciton relaxation yield for C_{60} bulk CT excitons. **c,** The η_{EQE} spectra for CdSe quantum dot (QD)-C545T planar photovoltaic devices in Supplementary Figure 9 versus QD thickness (42 and 67 nm).

Supplementary Note 1

Discussion for Supplementary Figure 5:

In many OPV designs, an exciton blocking layer (EBL) is not included on the ITO anode as it increases the bulk resistance and reduces device built-in field (E_{bi}). Many previous studies deposit the donor material directly on ITO or on deep work function buffer layers such as MoO_x [2-5]. Here, we compare three different anode contacts: ITO/mCP/donor, ITO/donor and ITO/MoO_x/donor for a SubPc-C_{60} PHJ. The E_{bi} within the device is expected to be highest for deep work function MoO_x and lowest for a more resistive EBL of mCP.

For the SubPc-C_{60} PHJ device with 10-nm-thick donor, the η_{CS} is found to be ~100% at short-circuit (Fig. 4a) even when a 7-nm-thick anode EBL of mCP is incorporated. As such, increasing E_{bi} will not further improve η_{CS} for this device. The impact of anode buffer layers and E_{bi} on exciton harvesting can be directly determined from changes in η_{IQE}. Figure S5a and b show the η_{EQE} and η_D ($\approx\eta_{IQE}$) of SubPc-C_{60} PHJ devices with different anode contacts. Compared to a device with an mCP EBL, the devices with bare ITO and MoO_x show lower η_{EQE} and η_D for the SubPc absorption dominant region. This suggests that the ITO and MoO_x surfaces can quench SubPc excitons and reduce photocurrent, consistent with the observation in Supplementary Figure 1. A lower L_D will be extracted assuming these interfaces are exciton reflecting when using previous charge carrier-based measurements. For the C_{60} absorption dominant region, the devices with bare ITO and MoO_x show an increase in η_{EQE} and η_D compared to the device with an mCP EBL. As we only varied the anode-donor interface, donor-acceptor and acceptor-cathode interfaces remain the same. Exciton diffusion is not expected to change within C_{60} layer based on the simulation[1]. As such, the mechanism for more efficient exciton harvesting is likely to be enhanced exciton bulk-ionization.

To isolate the role of bulk-ionization in exciton harvesting, a 5-nm-thick 2,2',2"-(1,3,5-benzinetriyl)-tris(1-phenyl-1-H-benzimidazole) TPBi interlayer is inserted between SubPc and C_{60}, to frustrate charge transfer (Supplementary Figure 5c). As no exciton dissociating interface is available, the only carrier generation pathway is bulk ionization by E_{bi}. Figure S5d shows the η_{EQE} of SubPc-TPBi-C_{60} PHJ devices with different anode contacts. These devices show increased η_{EQE} with E_{bi} and negligible photoresponse for λ > 550 nm, similar to the photoresponse of C_{60} Schottky OPVs[6]. This suggests that the free carriers are directly generated from high energy bulk CT excitons in C_{60} and the contribution from the lowest energy Frenkel excitons are negligible for both SubPc and C_{60}. The interlayer device with an mCP EBL shows very low η_{EQE} (~1% at λ = 400 nm), which verifies the assumption for η_{IQE}-based L_D measurement: excitons are only dissociated at D-A interface. However, the interlayer device with deep work function MoO_x has much higher η_{EQE} (~10 % for the maximum), close to 20% of the bilayer device η_{EQE} in the C_{60} dominant absorption region. In this case, the recombination losses and η_{CS} of this device are no longer identical for donor and acceptor materials due to the multiple sources for free carrier generation.

Supplementary Note 2

Discussion for Supplementary Figure 6:

As the devices described in the main text for the measurement of L_D are designed to avoid exciton dissociation everywhere but at the D-A interface, a comparison between these and conventional devices permits a quantitative probe of all quenching and dissociation pathways. Here, we fully decouple the various exciton relaxation pathways in a conventional device without an mCP anode buffer layer (Supplementary Figure 6a), which is the same device used in Supplementary Figure 5a.

The shaded areas in Supplementary Figure 6c and 6d denote the relative importance of each pathway. To realize this decoupling, we first determine the ITO-SubPc boundary condition for exciton quenching using the donor η_{EQE} at λ = 590 nm (~54%) in Supplementary Figure 6b (solid black line) and the intrinsic L_D of SubPc extracted using the methods described in the main text. This boundary condition allows us to simulate the theoretical donor η_{EQE} in the absence of exciton loss at the ITO-SubPc interface (dashed red line). Comparing the solid black and dashed red lines offers a measure of excitons lost at ITO-SubPc interface. The difference between the dashed red line and the solid red line (calculated η_A) is the fraction of donor excitons lost to natural decay.

The same analysis may also be performed for the acceptor, using the extracted acceptor η_{EQE} in Supplementary Figure 6b. As with Supplementary Figure 5, the photocurrent contribution from the bulk-ionization of C_{60} CT excitons can be determined from interlayer devices (Supplementary Figure 5c). Indeed, subtraction of the interlayer device η_{EQE} (red line, Supplementary Figure 5d) from the overall acceptor η_{EQE} (black line, Supplementary Figure 6d) yields the contribution of Frenkel excitons to the photoresponse (dashed black line, Supplementary Figure 6d). With this curve and the η_D of Frenkel excitons determined using the C_{60} L_D, the theoretical η_{EQE} of C_{60} can be derived (dashed blue line, Supplementary Figure 6d), assuming all Frenkel excitons are dissociated at the D-A interface. The difference between this curve and the calculated acceptor absorption efficiency (η_A, blue line, Supplementary Figure 6d) yields the exciton loss from CT exciton recombination.

Supplementary References

1. Pettersson L. A. A., Roman L. S. & Inganas O. Modeling photocurrent action spectra of photovoltaic devices based on organic thin films. *J. Appl. Phys.* **86,** 487-496 (1999).
2. Yi Y.*, et al.* The interface state assisted charge transport at the MoO_3/metal interface. *J. Chem. Phys* **130,** 094704 (2009).
3. Menke S. M., Luhman W. A. & Holmes R. J. Tailored exciton diffusion in organic photovoltaic cells for enhanced power conversion efficiency. *Nat. Mater.* **12,** 152-157 (2013).
4. Rand B. P., Burk D. P. & Forrest S. R. Offset energies at organic semiconductor heterojunctions and their influence on the open-circuit voltage of thin-film solar cells. *Phys. Rev. B* **75,** 115327 (2007).
5. Barito A.*, et al.* Universal design principles for cascade heterojunction solar cells with high fill factors and internal quantum efficiencies approaching 100%. *Adv. Energy Mater.* **4,** 1400216 (2014).
6. Hahn T.*, et al.* Role of intrinsic photogeneration in single layer and bilayer solar cells with C_{60} and PCBM. *J. Phys. Chem. C* **120,** 25083-25091 (2016).
